# Tight junction protein occludin is an internalization factor for SARS-CoV-2 infection and mediates virus cell-to-cell transmission

**DOI:** 10.1073/pnas.2218623120

**Published:** 2023-04-17

**Authors:** Jialin Zhang, Wenyu Yang, Sawrab Roy, Heidi Liu, R. Michael Roberts, Liping Wang, Lei Shi, Wenjun Ma

**Affiliations:** ^a^Department of Veterinary Pathobiology, College of Veterinary Medicine, University of Missouri, Columbia, MO 65211; ^b^Department of Molecular Microbiology and Immunology, School of Medicine, University of Missouri, Columbia, MO 65211; ^c^Division of Animal Sciences, College of Agriculture, Food, & Natural Resources, University of Missouri, Columbia, MO 65211; ^d^Christopher S Bond Life Sciences Center, University of Missouri, Columbia, MO 65211

**Keywords:** SARS-CoV-2, cell-to-cell transmission, occludin, internalization, SARS-CoV-2 variants

## Abstract

Severe acute respiratory syndrome coronavirus 2 (SARS-CoV-2) spreads efficiently by spike-mediated, direct cell-to-cell transmission. However, the underlying mechanism is poorly understood. Herein, we demonstrate that the tight junction protein occludin (OCLN) is critical to this process. SARS-CoV-2 infection alters OCLN distribution and expression and causes syncytium formation that leads to viral spread. OCLN knockdown fails to alter SARS-CoV-2 binding but significantly lowers internalization, syncytium formation, and transmission. OCLN overexpression also has no effect on virus binding but enhances virus internalization, cell-to-cell transmission, and replication. OCLN directly interacts with the SARS-CoV-2 spike, and the endosomal entry pathway is involved in OCLN-mediated cell-to-cell fusion rather than in the cell surface entry pathway. All SARS-CoV-2 strains tested (prototypic, alpha, beta, gamma, delta, kappa, and omicron) are dependent on OCLN for cell-to-cell transmission, although the extent of syncytium formation differs between strains. We conclude that SARS-CoV-2 utilizes OCLN as an internalization factor for cell-to-cell transmission.

## Significance

Although initial infection by Severe acute respiratory syndrome coronavirus 2 (SARS-CoV-2) occurs via a cell surface entry pathway through its spike protein binding to the angiotensin-converting enzyme 2 (ACE2) receptor, subsequent spread largely involves direct cell-to-cell transmission and syncytium formation, processes in which host factors required remain largely unknown. Here, we demonstrate that the tight junction protein occludin (OCLN) is a key internalization factor critical for these events. OCLN binds the spike protein and mediates cell-to-cell virus transmission. Cell-to-cell transmission of both prototypic and variant SARS-CoV-2 strains is reliant on spike protein binding to OCLN, even though the extent of subsequent syncytium formation is virus strain dependent. Our results will provide a better understanding of SARS-CoV-2 entry, transmission, and pathogenesis.

The causative agent of the COVID-19 pandemic starting in late December 2019 is a novel coronavirus, now named Severe acute respiratory syndrome coronavirus 2 (SARS-CoV-2) because of its close relationship and high sequence identity to SARS-CoV (1). SARS-CoV-2 is an enveloped, single-stranded, positive-sensed RNA virus that belongs to the genus Betacoronavirus in the family *Coronaviridae* (2). Compared with SARS-CoV and Middle East respiratory syndrome coronavirus (MERS-CoV), which mainly target human lungs leading to acute respiratory failure, the novel SARS-CoV-2 shows multiorgan tropisms in addition to the respiratory tract that can lead to severe multiple organ failures (3, 4). As of 16 February 2023, SARS-CoV-2 has caused more than 756 million human infections and approximately 6.8 million deaths worldwide (https://covid19.who.int/). Since its emergence, SARS-CoV-2 has evolved rapidly by acquiring mutations, resulting in multiple variants of concern that show more effective transmission than the original prototype isolate. This is especially true for the omicron variants of SARS-CoV-2 that first emerged in November 2021. These are characterized by a large number of mutations within the spike protein (5), which have provided the variants greater capability of evading neutralizing antibodies induced by vaccination (6). Omicron variants are presently the dominant forms circulating in the United States and infect individuals who have been fully vaccinated including those who have received an additional booster (7). This feature of omicron and its ability to spread rapidly most likely underpins the most recent wave of SARS-CoV-2 infection around the world (5).

Binding to the target cells is the first step to initiate virus efficient infection. SARS-CoV-2 spike (S) binds to the primary receptor, angiotensin-converting enzyme 2 (ACE2), to mediate virus attachment and entry into the host cells. To initiate virus infection, the S protein needs to be cleaved into S1 and S2 at the S1/S2 boundary. The S1 subunit containing the receptor binding domain (RBD) is responsible for binding to ACE2, while the S2 subunit mediates the fusion of the viral envelope with the cell membrane and release of the viral genome into the cytoplasm (8). As a result of the cleavage of the S protein at the S1/S2 boundary, SARS-CoV-2 infection in humans leads to the formation of infected cell syncytia in lung tissues, which has previously been noted to be associated with SARS-CoV-2 pathogenicity (9). This induction of fusion of virus-infected cells with neighboring cells and the creation of syncytia contribute to increased viral transmission through direct cell-to-cell spreading (10–12), as also documented for several other coronaviruses, such as MERS-CoV and bronchitis virus (13, 14). Indeed, such cell-to-cell transmission contributes to virus pathogenesis by allowing SARS-CoV-2 to escape vaccine-induced neutralization antibodies (15). An homologous strategy has also been used by hepatitis C virus (16) and HIV (17). However, the underlying mechanisms of S protein–induced cell-to-cell transmission remain poorly understood, and the cellular factors involved in cell-to-cell transmission have not been identified.

SARS-CoV-2 targets the respiratory tract and efficiently infects airway epithelial cells (18). An essential component of the barrier function of epithelial surfaces to pathogens is the presence of intercellular structures named tight junctions (TJs) that are formed between neighboring cells and regulate passage of ions and other small solutes (19). TJs are associated with complexes that mediate cell adhesion and contain the protein occludin (OCLN), which has been identified as an essential host factor for entry of coxsackie B virus (20), rotavirus (21), human hepatitis C virus (22), West Nile virus (23), and porcine epidemic diarrhea virus (PEDV) (24). Considering that virus-induced cell fusion requires a breakdown of the barrier between cells, we speculate that TJ proteins may be involved in allowing SARS-CoV-2 to spread through cell-to-cell transmission. Here, we have identified a previously unrecognized role of OCLN as an internalization factor in SARS-CoV-2 entry and cell-to-cell transmission.

## Results

### SARS-CoV-2 Infection Alters the Expression of OCLN in Target Cells.

**Fig. 1.**
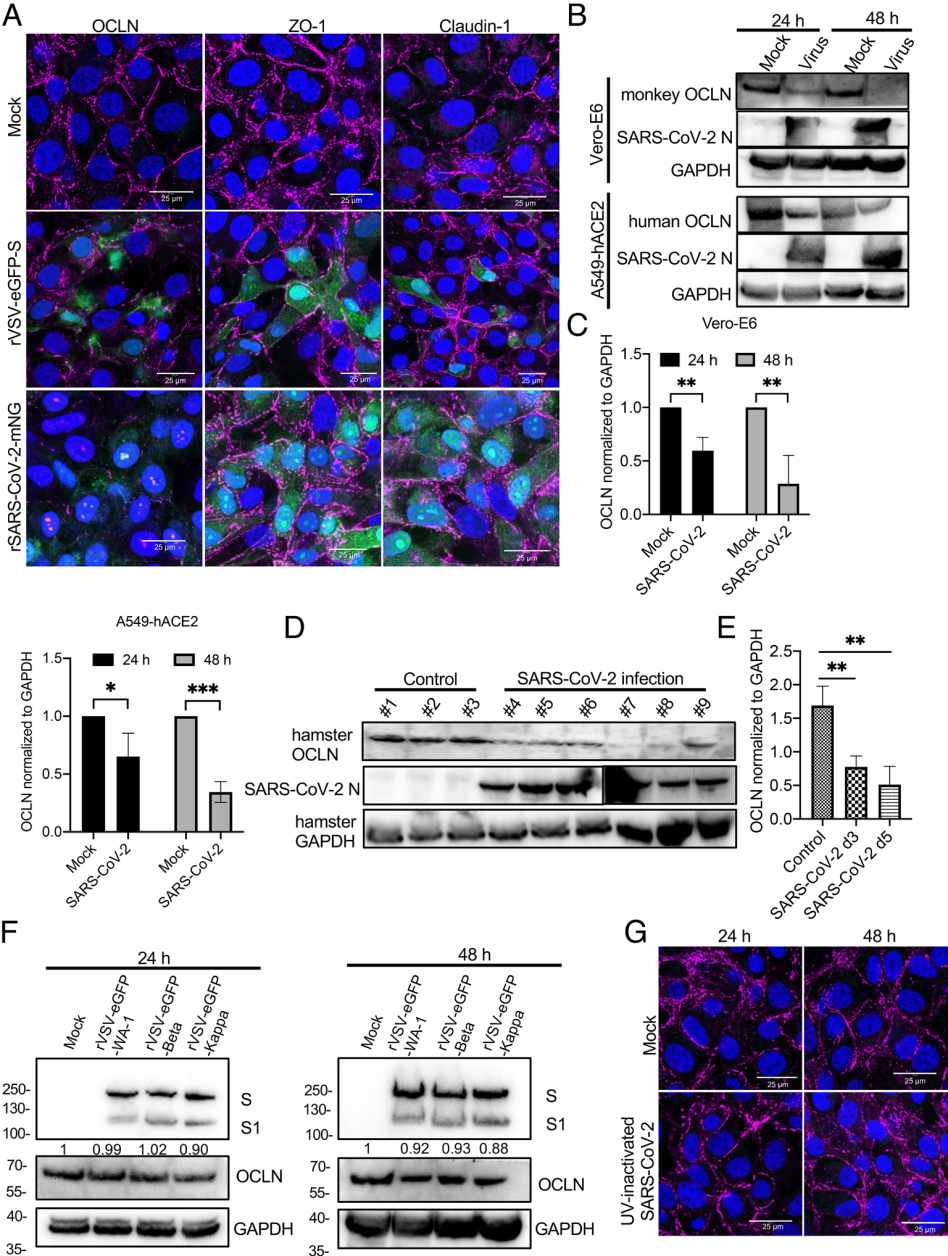
SARS-CoV-2 infection affects OCLN expression. (*A*) Vero-E6 cells were infected with rVSV-eGFP-S or SARS-CoV-2-mNG virus at an MOI of 0.1 and then fixed and stained with OCLN, ZO-1, and Claudin-1 antibodies to detect each protein expression and distribution 24 h postinfection by the confocal assay. (*B*) Vero-E6 cells and A549-hACE2 cells were infected with SARS-CoV-2 at an MOI of 0.01 at indicated different time points. Subsequently, cells were collected, and lysates were prepared for western blot by normalizing to GAPDH. (*C*) OCLN expression after SARS-CoV-2 infection was quantified by western blot (three independent experiments). (*D*) OCLN expression in lung tissues of hamsters mock-infected at day 3 (#1, #2, and #3) or infected with SARS-CoV-2 at day 3 (#4, #5, and #6) and day 5 (#7, #8 and #9) postinfection was determined by western blot. (*E*) OCLN expression was quantified for the results of western blot by normalizing to GAPDH. (*F*) Vero-E6 cells were infected with rVSV-eGFP-WA-1, rVSV-eGFP-Beta, or rVSV-eGFP-Kappa virus at an MOI of 0.1. Forty-eight hours postinfection, the cells were collected and prepared for western blot. (*G*) Vero-E6 cells were mock-infected or infected with an UV-inactivated SARS-CoV-2. Cells were fixed, and IFA was performed to detect OCLN. Nuclei were stained by 4′,6-diamidino-2-phenylindole (DAPI).

To evaluate the effect of SARS-CoV-2 infection on TJ proteins, we first determined the distribution of OCLN, ZO-1, and Claudin-1 in Vero-E6 cells infected with a recombinant vesicular stomatitis virus (rVSV) expressing eGFP and SARS-CoV-2 spike (rVSV-eGFP-S) or recombinant SARS-CoV-2 expressing the mNeonGreen gene (SARS-CoV-2-mNG). Both rVSV-eGFP-S and SARS-CoV-2-mNG infections were able to alter OCLN expression and distribution but provided no observable changes on ZO-1 and Claudin-1 (Fig. 1*A*). As demonstrated by western blot, OCLN levels were also significantly decreased 24 h postinfection and undetectable 48 h postinfection in infected Vero-E6 cells, indicating that OCLN was degraded following SARS-CoV-2 infection (Fig. 1 *B* and *C*). This result was also confirmed in human A549-hACE2 cells infected with SARS-CoV-2 where OCLN expression was also reduced relative to mock-infected cells (Fig. 1 *B* and *C*). Furthermore, OCLN expression was also significantly decreased in the lungs of infected hamsters 3 and 5 d postinfection in contrast to mock-infected animals (Fig. 1 *D* and *E*). These results indicate that SARS-CoV-2 infection significantly reduces OCLN expression and leads to its destruction in vitro and in vivo. To understand the underlying mechanisms, rVSV-eGFP viruses bearing different spikes (rVSV-eGFP-WA-1, rVSV-eGFP-Beta, and rVSV-eGFP-Kappa) or UV-inactivated SARS-CoV-2 were used to infect Vero-E6 cells to determine whether OCLN expression was altered. No significant differences in OCLN expression in infected cells were observed among rVSV-eGFP viruses bearing different spikes (Fig. 1*F*) and between mock-infected and UV-inactivated SARS-CoV-2 infected cells (Fig. 1*G*). These results demonstrated that the changes in OCLN expression were SARS-CoV-2 infection dependent.

### OCLN Is Required for SARS-CoV-2 Infection.

**Fig. 2.**
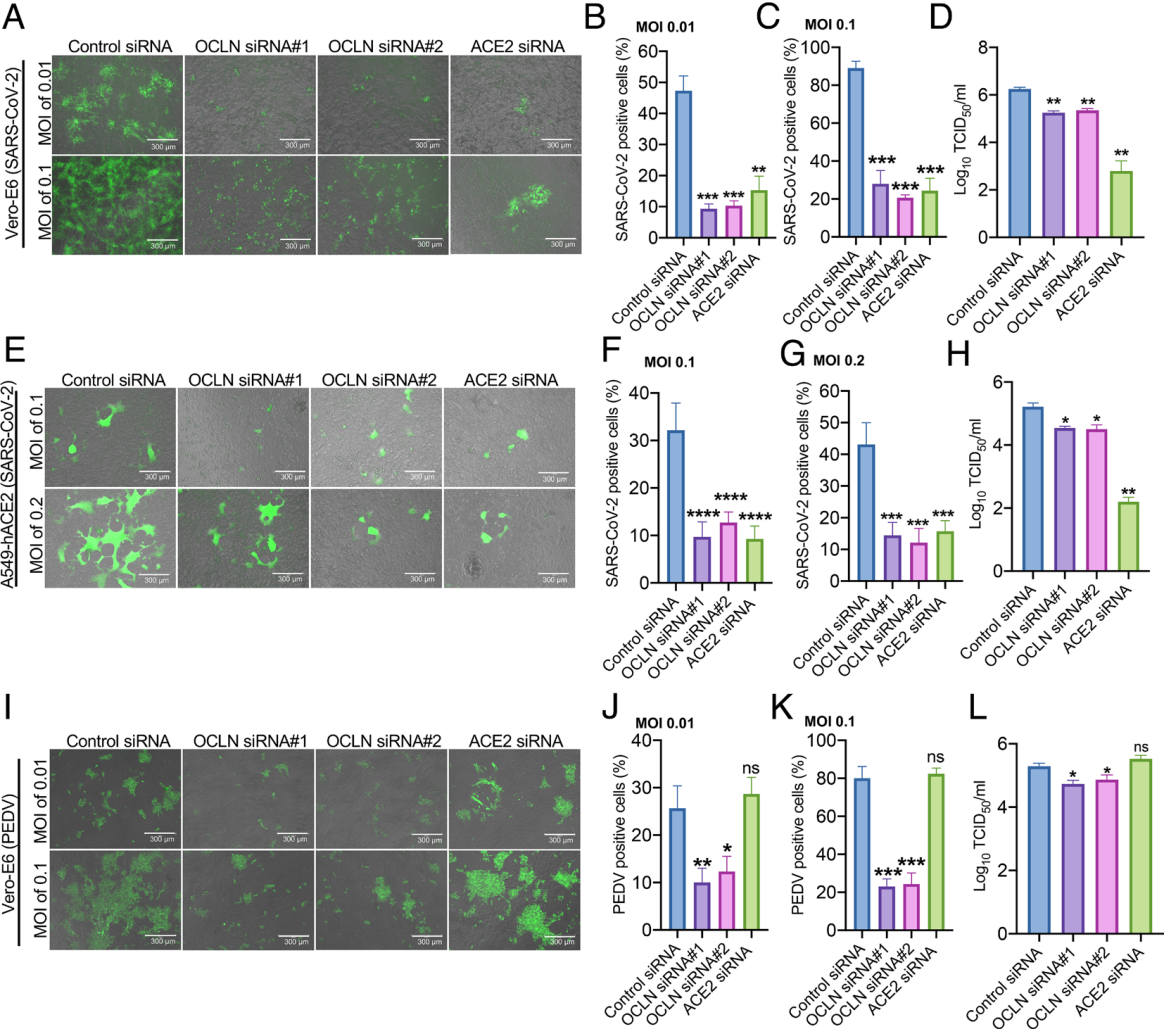
OCLN knockdown strongly inhibits SARS-CoV-2 infection and replication. (*A–D*) Vero-E6 cells were transfected with control siRNA, OCLN siRNAs, or ACE2 siRNA and then infected with SARS-CoV-2-mNG at indicated MOIs. Forty-eight hours postinfection, fluorescence images (*A*) representative of three independent experiments were captured (scale bars, 300 μm), and SARS-CoV-2-positive cells (*B* and *C*) were analyzed for each MOI by analyzing at least 10 different fields. Virus titer (*D*) was determined in three independent experiments after infection at an MOI of 0.01. (*E*–*H*) A549-hACE2 cells were transfected with control siRNA, OCLN siRNAs, or ACE2 siRNA and then infected with SARS-CoV-2-mNG at indicated MOIs. Forty-eight hours postinfection, fluorescence images (*E*) representative of three independent experiments were captured (scale bars, 300 μm), and SARS-CoV-2-positive cells (*F* and *G*) were analyzed for each MOI. At least 10 different fields analyzed for each group. Virus titer (*H*) from three independent experiments was determined after cells were infected at an MOI of 0.1. (*I*–*L*) Vero-E6 cells were transfected with control siRNA, OCLN siRNA, or ACE2 siRNA and then infected with PEDV at indicated MOIs. Forty-eight hours postinfection, fluorescence images (*I*) representative of three independent experiments were captured (scale bars, 300 μm), and PEDV-positive cells (*J* and *K*) were analyzed for each MOI. At least 10 different fields analyzed for each group. The virus titer (*L*) from three independent experiments was determined after cells were infected at an MOI of 0.1.

To determine whether the OCLN is required for SARS-CoV-2 infection in permissive cells, Vero-E6 cells were transfected with two specific small interfering RNAs (siRNAs) targeted to OCLN, whose subsequent loss of expression was confirmed by the immunofluorescence assay (IFA) (*SI Appendix*, Fig. S1*A*), western blot (*SI Appendix*, Fig. S1*B*), and RT-qPCR (*SI Appendix*, Fig. S1*C*). Importantly, OCLN knockdown had no effect on the expression of ACE2 as determined by RT-qPCR of its transcript levels and western blot for ACE2 protein in both Vero-E6 cells and A549-hACE2 cells (*SI Appendix*, Fig. S1 *B* and *C*). The involvement of OCLN in viral infection and replication was further examined by comparing virus responses of cells in which OCLN expression had been reduced by siRNA silencing and controls in which ACE2 knockdown had been performed. Both were infected with the SARS-CoV-2-mNG virus. Cells infected with PEDV served as the control. ACE2 knockdown was confirmed by RT-qPCR and western blot (*SI Appendix*, Fig. S2 *A* and *B*). As shown in Fig. 2*A*, there was a significant reduction (average 80.4% and 78.3%) of cells positive for SARS-CoV-2 after infection at MOIs of 0.01 and 0.1 in OCLN knockdown Vero-E6 cells compared to control siRNA–treated cells. A comparable decrease in positive cells was observed in the ACE2 knockdown cells (average 67.8% and 73%) (Fig. 2 *B* and *C*). The lower number of virus-positive cells induced by two OCLN-specific siRNAs correlated with a significantly lower level of virus production (average reduction 89.5%), while ACE2 silencing led to an even more effective reduction of SARS-CoV-2 titer (97.1%) (Fig. 2*D*). To further confirm the results observed in Vero-E6 cells, we examined the SARS-CoV-2 infection and yield in a human lung cell line (A549-hACE2). Both OCLN and ACE2 knockdowns significantly reduced SARS-CoV-2 infection (average 67% reduction for OCLN siRNA and 70% reduction for ACE2 siRNA at an MOI of 0.1) (Fig. 2 *E*–*G*). Again, however, ACE2 knockdown resulted in much lower virus production (96.7%) relative to that observed with OCLN knockdown (85.2% and 86.0% for OCLN siRNA#1 and OCLN siRNA#2, respectively) (Fig. 2*H*), values that were consistent with the values observed on Vero-E6 cells after OCLN knockdown. As expected, OCLN knockdown in Vero-E6 cells also dramatically reduced the extent of PEDV infections (average 58.3% and 70.6% for OCLN siRNA#1 and OCLN siRNA#2, respectively) and virus titers (79.4% reduction at an MOI 0.01), while ACE2 knockdown did not (Fig. 2 *I*–*L*). Additionally, we also generated OCLN knockout (KO) Vero-E6 cell line. Its depletion was validated by IFA and western blot (*SI Appendix*, Fig. S3 *A* and *B*) to further confirm siRNA knockdown results. As shown in *SI Appendix*, Fig. S3 *C* and *D*, OCLN KO significantly reduced SARS-CoV-2-mNG infection compared to the wild-type Vero-E6 cells (72% reduction), which is consistent with the results obtained in OCLN siRNA knockdown Vero-E6 cells (62.5% reduction). Together, these results demonstrate that OCLN knockdown leads to reduced susceptibility of permissive cells to SARS-CoV-2 infection.

### OCLN Interacts with SARS-CoV-2 S1 and ACE2, While OCLN Silencing Inhibits Virus Internalization via Micropinocytosis.

**Fig. 3.**
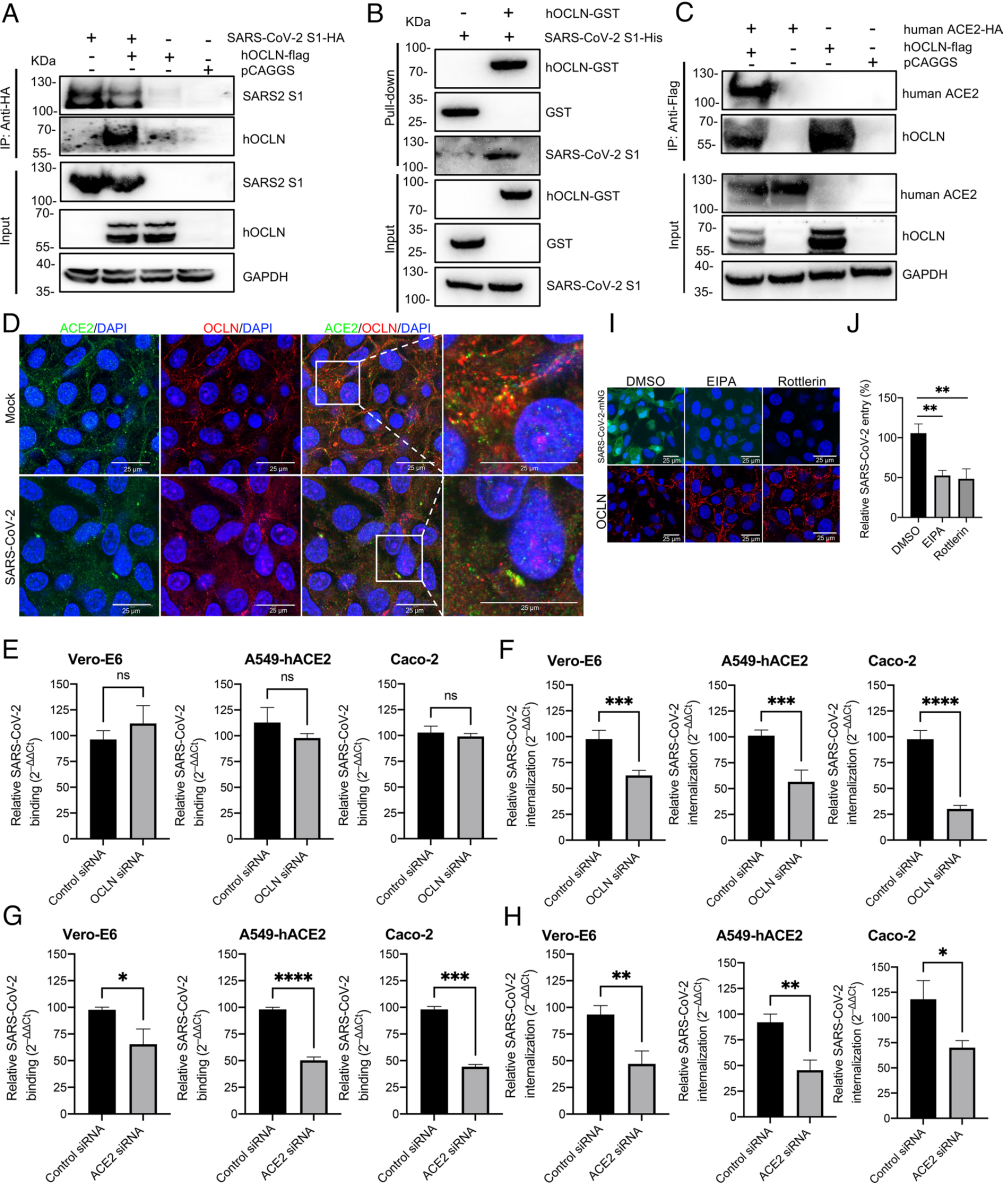
SARS-CoV-2 S protein directly interacts with OCLN and SARS-CoV-2 internalization but not virus binding requires OCLN. (*A*) SARS-CoV-2 S1 tagged with HA and human OCLN plasmids tagged with Flag were cotransfected into 293T cells. At 72 h posttransfection, the cell lysate was harvested. Co-IP was performed using an anti-HA antibody. The precipitated proteins were detected by western blot using antibodies against the HA and Flag tags. (*B*) For the GST pull-down assay, GST and GST-tagged hOCLN were expressed in *Escherichia coli* BL21(DE3) and purified with glutathione resin. The resin was incubated with purified SARS-CoV-2 His-S1 protein, and the bound proteins were detected by western blot using an anti-GST antibody and an anti-His antibody. (*C*) Interaction between OCLN and ACE2. Human ACE2 plasmid tagged with HA and human OCLN plasmid tagged with Flag were cotransfected into 293T cells. 72 h posttransfection, the cell lysate was harvested. Co-IP was performed by using anti-Flag antibody. The precipitated proteins were detected by western blot with antibodies against the HA and Flag tags. (*D*) Colocalization of OCLN and ACE2. Vero-E6 cells were mock-infected or infected with SARS-CoV-2 at an MOI of 0.1. 48 h postinfection, the cells were fixed. ACE2 and OCLN were detected by an anti-ACE2 and an anti-OCLN antibody. Images were acquired with a confocal microscope. (*E*) Virus binding assay: Vero-E6, A549-hACE2, or Caco-2 cells were infected with SARS-CoV-2 at an MOI of 1 at 4 °C for 1 h. Unbound viruses were removed by washing with cold phosphate buffered saline (PBS), and cells then treated with 1 mL TRIzol to extract RNA for RT-qPCR. (*F*) Virus internalization assay: Cells were transfected with OCLN or control siRNA and then infected with SARS-CoV-2 at an MOI of 1 at 4 °C for 1 h. Unbound viruses were removed with cold PBS. The cells were transferred to 37 °C for 1 h to allow virus internalization and then washed to remove bound virus on cell surface with acidic buffer. The cells were lysed, and RNA was prepared for RT-qPCR. ACE2 knockdown with specific siRNA was used as a positive control for virus binding assay (*G*), and virus internalization assay (*H*) was performed. (*I*) Vero-E6 cells were treated with micropinocytosis inhibitors, EIPA, or rottlerin for 1 h prior to SARS-CoV-2-mNG infection. Forty-eight hours postinfection, virus infection was observed through GFP signals, and OCLN distribution was detected with IFA by using an anti-OCLN antibody. (*J*) Vero-E6 cells were pretreated with EIPA or rottlerin and then infected with SARS-CoV-2 at 4 °C for 1 h. Unbound viruses were removed with the cold PBS. The cells were transferred to 37 °C for 1 h to allow virus internalization and then washed to remove bound virus on cell surface with acidic buffer. The cells were lysed, and RNA was prepared for RT-qPCR to determine virus entry.

The first step that initiates efficient infection of SARS-CoV-2 is to bind to ACE2 via the S1 protein RBD (25). We hypothesize that SARS-CoV-2 spike protein subsequently interacts with OCLN to mediate virus entry. To test this hypothesis, plasmids expressing HA-tagged SARS-CoV-2 S1 and Flag-tagged human OCLN (hOCLN) were cotransfected into 293T cells to perform the coimmunoprecipitation (co-IP) assay. Immunoblotting for S1-HA demonstrated that hOCLN protein interacts with SARS-CoV-2 S1 protein specifically (Fig. 3*A*). To determine whether a direct interaction occurs between S1 and hOCLN, either purified GST or GST-tagged hOCLN was incubated with the His-tagged S1 purified from the HEK293T cells transfected with pCAGGS-S1-His. In the GST pull-down assay, S1-His was pulled down by GST-hOCLN, indicating that S1 directly interacts with hOCLN (Fig. 3*B*). To show whether OCLN also interacts with the ACE2, we performed the co-IP assay by transfecting plasmids expressing HA-tagged human ACE2 and Flag-tagged hOCLN into 293T cells (Fig. 3*C*) and observed the interaction between the two proteins. We also used confocal microscopy to determine whether or not they colocalized in Vero-E6 cells that had either been mock-infected or infected with SARS-CoV-2. Results were again consistent with an interaction between hOCLN and hACE2 within the cytoplasm after they had been infected with the live virus (Fig. 3*D*). However, a colocalization signal between hOCLN and hACE2 was also detected in mock-infected as well as SARS-CoV-2-infected cells (Fig. 3*D*). Taken together, these results demonstrate that OCLN can interact with SARS-CoV-2-S1 directly and has an interaction with ACE2, suggesting that a complex formed among three molecules mediates virus entry and infection.

Interestingly, OCLN siRNA knockdown did not influence SARS-CoV-2 binding to Vero-E6, A549-hACE2, and Caco-2 cells (Fig. 3*E*) but significantly reduced virus internalization (Fig. 3*F*), demonstrating that OCLN is not required for viral attachment but critical for virus internalization. In contrast, ACE2 knockdown significantly diminished virus binding and internalization (Fig. 3 *G* and *H*). Since OCLN directly interacts with the S1 protein of SARS-CoV-2 (Fig. 3*B*) and is required for virus internalization, we hypothesized that it serves as a scaffold between cells to promote virus internalization postSARS-CoV-2 S1 binding to OCLN. To test this hypothesis, Vero-E6 cells were treated with a micropinocytosis inhibitor EIPA [5-(N-ethyl-N-isopropyl) amiloride] prior to SARS-CoV-2-mNG infection. EIPA pretreatment significantly reduced SARS-CoV-2 infection and abolished the degradation of OCLN compared with the DMSO-treated control group (Fig. 3*I*). A second micropinocytosis inhibitor rottlerin had a similar effect to EIPA and was even more effective at preventing SARS-CoV-2-mNG infection (Fig. 3*I*). Additionally, these two inhibitors significantly inhibited SARS-CoV-2 entry (average reduction 52.4%) as determined by RT-qPCR (Fig. 3*J*). These results implicate micropinocytosis in the internalization of the SARS-CoV-2/OCLN complex.

### OCLN C-Terminal Is Responsible for Mediating SARS-CoV-2 Internalization and Cell-to-Cell Transmission.

**Fig. 4.**
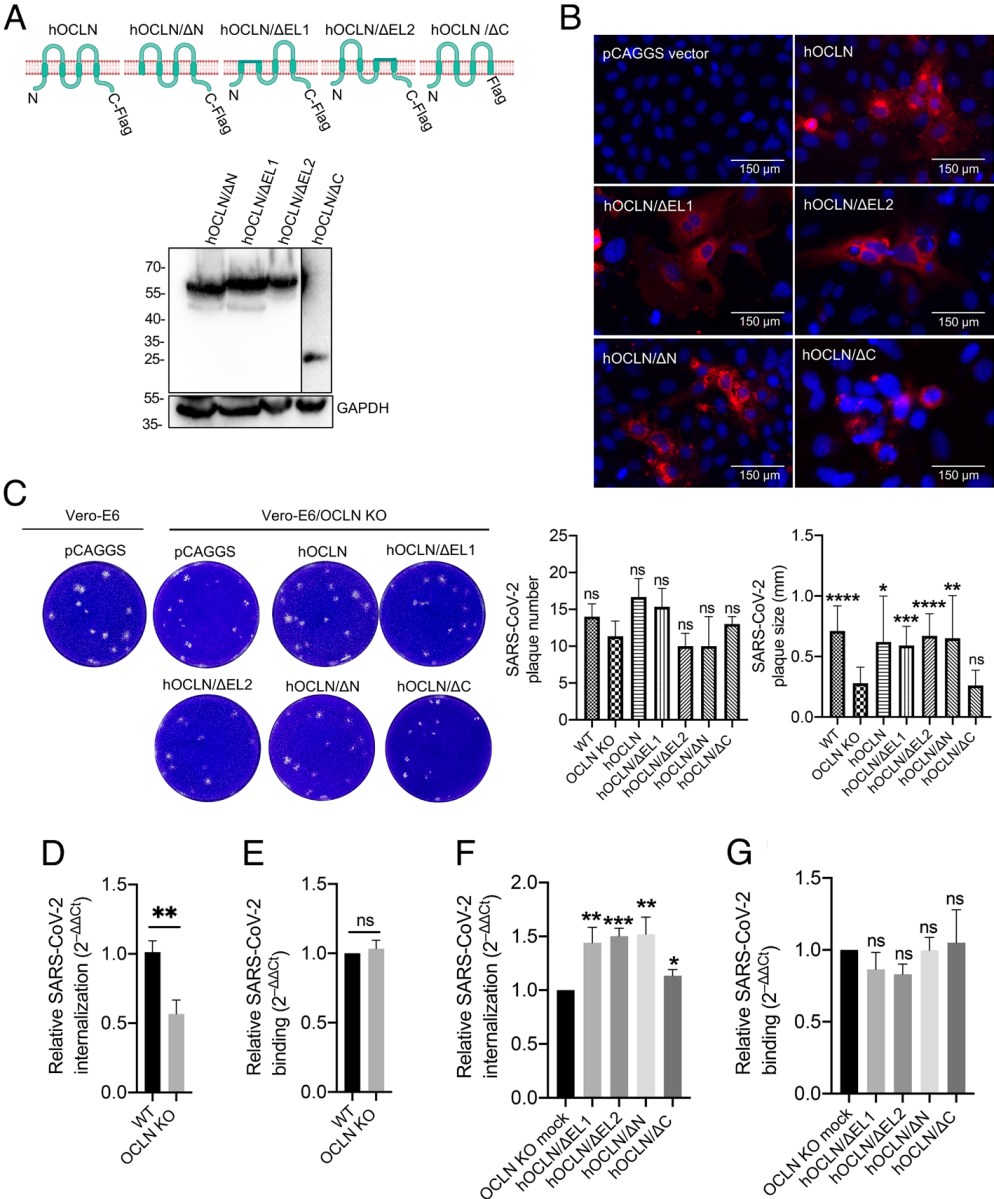
OCLN C-terminal is responsible for mediating SARS-CoV-2 cell-to-cell transmission and internalization. (*A*) Human OCLN and its four deletion constructs with a Flag tag in the C-terminal were constructed, and their expressions were confirmed by western blot. (*B*) OCLN constructs were transfected into Vero-E6 OCLN KO cell line, and protein expression was determined by IFA using the Flag antibody. (*C*) OCLN constructs or pCAGGS vector were transfected into the Vero-E6 OCLN KO cell line using the wild-type Vero-E6 cells as controls. Forty-eight h posttransfection, the cells were infected with SARS-CoV-2 at an MOI of 0.001, and the plaque assay was performed to determine plaque numbers and plaque size. (*D–G*) Virus internalization and binding assay: Vero-E6 OCLN KO or wild-type Vero-E6 cells were infected with SARS-CoV-2 at an MOI of 1 to determine virus internalization (*D*) and virus binding (*E*), which were measured by RT-qPCR. Vero-E6 OCLN KO cells were transfected with each OCLN deletion construct for 48 h and then infected with SARS-CoV-2 at an MOI of 1. Virus internalization (*F*) and virus binding (*G*) were determined by RT-qPCR.

As a component of the TJ of polarized cells, OCLN exists as a four-helix transmembrane protein that possesses a long intracellular N-terminal domain, two extracellular loops (EL1 and EL2), a short intracellular loop linking EL1 and EL2, and a long cytoplasmic tail (C-terminal) (26). To determine which domain is critical for virus internalization and cell-to-cell transmission, four OCLN deletion constructs, namely hOCLN/ΔC, hOCLN/ΔE1, hOCLN/ΔE2, and hOCLN/ΔN (Fig. 4*A*), were transfected into the OCLN KO Vero-E6 cell line. The expression of each was confirmed by western blot and IFA with an anti-Flag antibody (Fig. 4 *A* and *B*). As shown in Fig. 4*B*, C-terminal deletion of OCLN significantly altered its expression and distribution among cells compared to other deletion constructs. The impacts of these deletions on SARS-CoV-2 transmission between cells were measured by plaque assay and revealed that overexpression of all four deletion constructs in OCLN KO cells failed to affect plaque numbers. However, overexpression of hOCLN/ΔC resulted in small-sized plaques comparable to those formed in OCLN KO cells. By contrast, large-sized plaques comparable in diameter to those found in wild-type Vero-E6 cells were observed cells overexpressing the other three constructs, (Fig. 4*C*). We then used the same Vero-E6 OCLN KO cell line to determine whether SARS-CoV-2 binding and internalization were affected. Results were consistent with the findings in OCLN siRNA knockdown cells, i.e., OCLN KO significantly reduced virus internalization but not virus binding (Fig. 4 *D* and *E*). Moreover, overexpression of any of the four deletion constructs had no effect on virus binding but could significantly enhance virus internalization. Of note, however, overexpression of hOCLN/ΔC had much less of an effect on virus internalization than the other three constructs (Fig. 4 *F* and *G*).

Furthermore, we conducted an antibody blocking assay with an OCLN polyclonal antibody that targets the two extracellular EL1 and EL2 loops using the bovine serum albumin (BSA) treatment as a negative control. While plaque size was unaffected, plaque numbers were significantly reduced when a high concentration of antibody was used in contrast to BSA-treated groups (*SI Appendix*, Fig. S4). Taken together, these results indicate that the C-terminal of OCLN plays a critical role in mediating SARS-CoV-2 cell-to-cell transmission and internalization, while the exposed EL1 and EL2 loops play no significant role.

### Overexpression of OCLN Facilitates SARS-CoV-2 Infection and Replication.

**Fig. 5.**
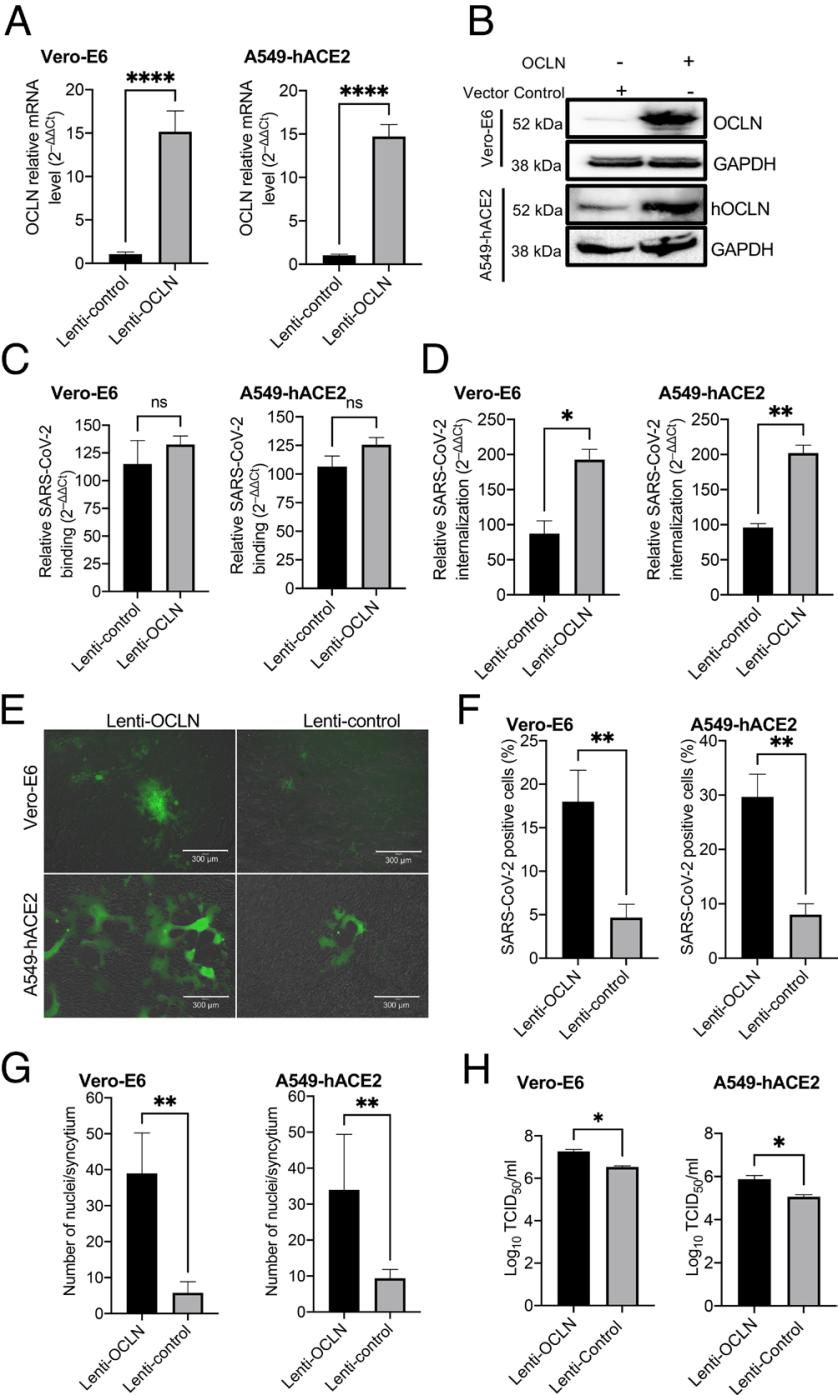
OCLN overexpression facilitates SARS-CoV-2 infection. (*A–D*) Vero-E6 or A549-hACE2 cells were infected with lentivirus expressing hOCLN. After 48 h, cells were prepared for RT-qPCR (*A*) and western blot (*B*) to detect OCLN expression at mRNA and protein levels. In the control group, because of the cross-reaction of OCLN antibody with both monkey and human OCLN, monkey OCLN also can be detected by western blot. (*C*) Virus binding and (*D*) virus internalization assays on Vero-E6 and A549-hACE2 cells were performed as described above. (*E–H*) Vero-E6 or A549-hACE2 cells in which OCLN was overexpressed were infected with SARS-CoV-2-mNG virus at an MOI of 0.01 for 24 h, and images (*E*) were captured with EVOS™ M5000 Imaging System; SARS-CoV-2-mNG-positive cells (*F*) and cell numbers for each syncytium (*G*) were calculated, and virus yield (*H*) was determined by the TCID_50_ assay.

A549-hACE2 and Vero-E6 cells were infected with lentivirus expressing hOCLN. After 48 h transfection, OCLN had become overexpressed in both cell lines at the level of both its mRNA (Fig. 5*A*) and protein (Fig. 5*B*). OCLN overexpression had no effect on virus binding (Fig. 5*C*) but significantly increased virus internalization (Fig. 5*D*). Furthermore, OCLN overexpression significantly enhanced syncytium formation in each cell line (Fig. 5*E*), as evident from the increased numbers of infected cells in the cultures (average 74.4% and 73.0%) and number of nuclei per syncytium (average 85.3% and 75.6%) (Fig. 5 *F* and *G*). There was also a significantly higher virus titer detected in both OCLN-overexpressing Vero-E6 and A549-hACE2 cultures (Fig. 5*H*). Additionally, we also tested whether overexpression of mouse OCLN (mOCLN) influenced virus cell-to-cell transmission, virus internalization, and binding in the OCLN KO Vero-E6 cell line. mOCLN expression could be confirmed by IFA (*SI Appendix*, Fig. S5*A*) and significantly impacted SARS-CoV-2 cell-to-cell transmission as evidenced by the increased plaque size with no effect on plaque numbers (*SI Appendix*, Fig. S5 *B* and *C*). Consistent with our earlier observations on hOCLN expression, mOCLN overexpression resulted in increased virus internalization rather than virus binding (*SI Appendix*, Fig. S5*D*). Taken together, these data demonstrate that OCLN overexpression significantly enhances infection and replication of SARS-CoV-2 in permissive cells, again emphasizing that OCLN plays an important role in virus-induced cell-to-cell fusion and virus cell-to-cell transmission.

### OCLN Knockdown Inhibits SARS-CoV-2 S Protein-Induced Cell-to-Cell Fusion.

**Fig. 6.**
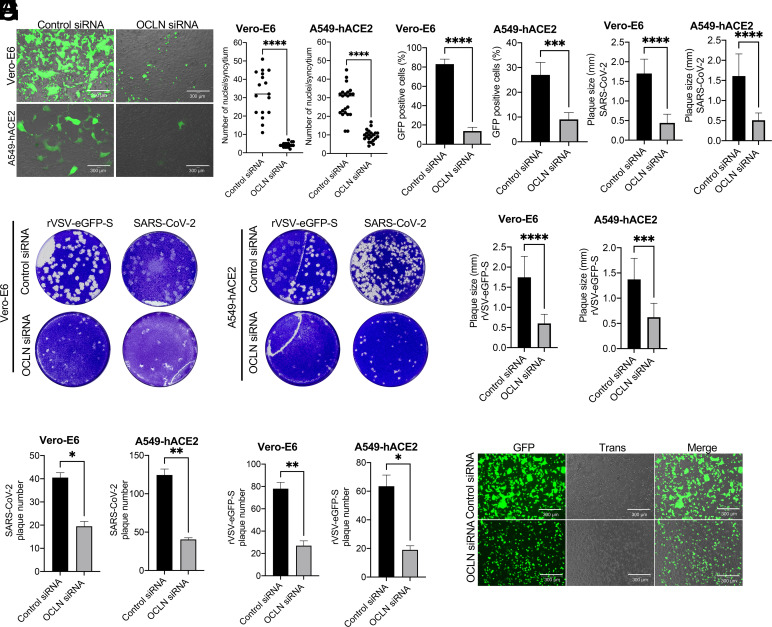
OCLN knockdown significantly inhibits spike protein–mediated cell-to-cell fusion. (*A–C*) Vero-E6 or A549-hACE2 cells were transfected with OCLN siRNA or control siRNA and infected with rVSV-eGFP-S virus at an MOI of 0.1 for 1 h followed by overlaying with 3% agarose. 72 h postinfection, images (*A*) were captured (scale bars, 300 μm); numbers of nuclei for each syncytium (*B*) (n > 15) and GFP-positive cells (*C*) (n >10 different fields for each group) were analyzed. (*D–H*) Vero-E6 cells transfected with OCLN siRNA or control siRNA were infected with rVSV-eGFP-S or SARS-CoV-2, respectively. Cells were washed and overlaid with 3% agarose to perform plaque assays (*D*). Plaque size (*E* and *F*) and plaque numbers (*G* and *H*) were analyzed with ImageJ software. (*I*) Vero-E6 cells were transfected with the SARS-CoV-2 S plasmid carrying a GFP marker. S protein–induced cell-to-cell fusion was observed by a fluorescence microscope.

A previous study has shown that the cleavage of S by furin protease is critical for S protein–mediated syncytium formation, which, in turn, contributes to increased virus transmission (10). Among the tools to investigate virus cell-to-cell transmission, rVSV expressing virus glycoprotein system is a powerful tool (27), which has been utilized to study the transmission of SARS-CoV-2 (15) and Ebola virus (28). To determine whether OCLN is critical for cell-to-cell fusion, the cells were infected with rVSV bearing SARS-CoV-2 spike and overlaid with 3% agarose to block virus infection by virus released into the culture medium. Based on GFP signals, OCLN knockdown resulted in significant inhibition of the ability of rVSV-eGFP-S virus to infect and replicate in Vero-E6 cells and A549-hACE2 cells relative to controls with a normal OCLN content (Fig. 6*A*), which is consistent with the findings observed with SARS-CoV-2-mNG infection (Fig. 2 *A* and *E*). OCLN silencing also significantly blocked S protein–mediated cell-to-cell fusion and reduced syncytium formation among infected cells (Fig. 6*B*), which is in agreement with less GFP-positive signals observed (Fig. 6*C*). Additionally, there were significant reductions in plaque formations and size (Fig. 6 *D*–*F*) and plaque numbers (Fig. 6 *G* and *H*) in OCLN knockdown Vero-E6 and A549-hACE2 cells infected with either SARS-CoV-2 or rVSV-eGFP-S virus. To exclude the possibility that the observed cell-to-cell fusion might have been caused by other structural proteins associated with the VSV, the plasmid encoding the fusion protein of SARS-CoV-2 spike and GFP was transfected into OCLN-silenced Vero-E6 cells. A reduced syncytial phenotype was observed compared to control cells (Fig. 6*I*). Taken together, OCLN knockdown dramatically inhibits SARS-CoV-2 cell-to-cell fusion and transmission.

### Endosomal Entry Pathway Involves OCLN-Mediated SARS-CoV-2 Cell-to-Cell Transmission.

**Fig. 7.**
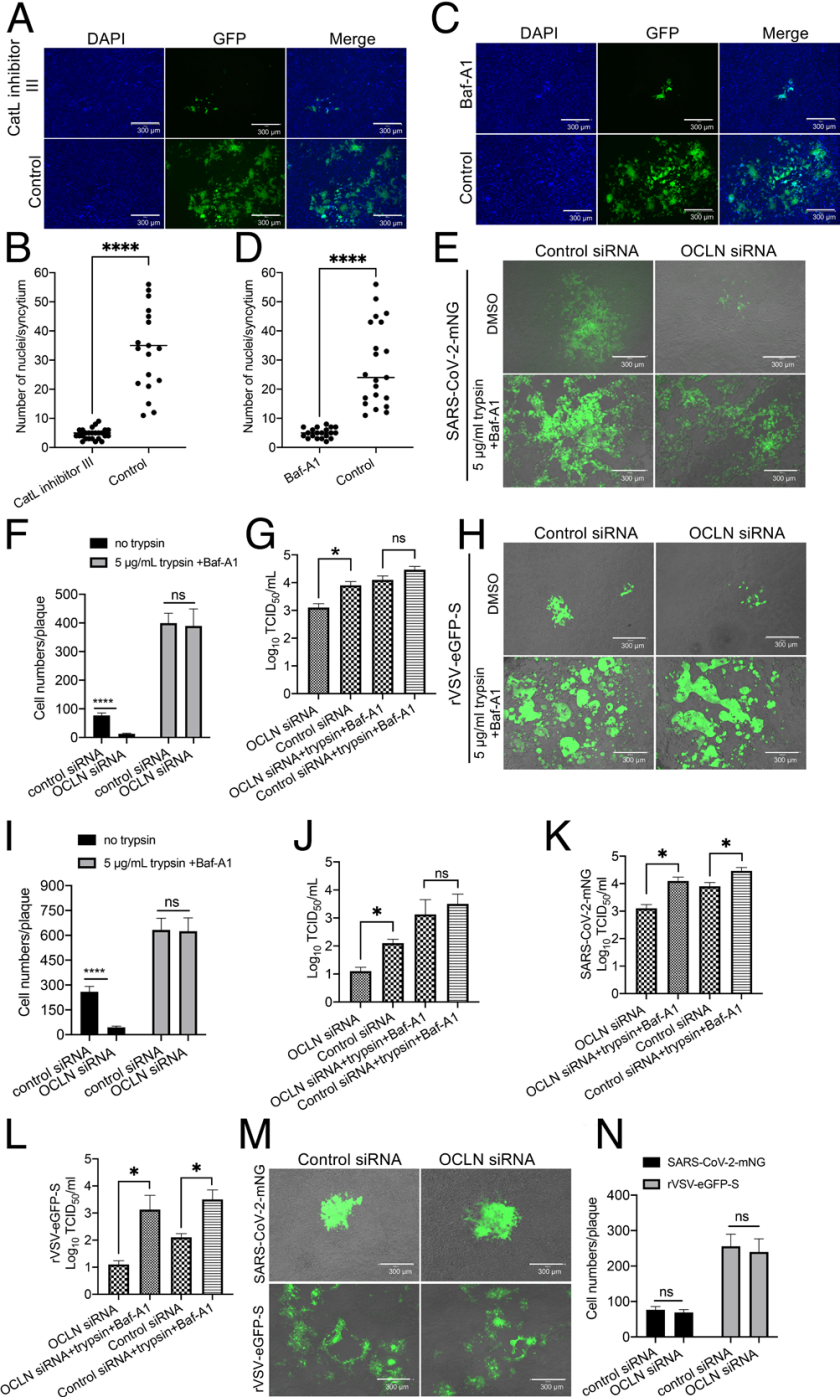
Endosomal entry pathway is involved in OCLN-mediated cell-to-cell fusion. (*A*–*D*) Vero-E6 cells were infected with rVSV-eGFP-S and then treated with CatL inhibitor III or Baf-A1, and virus-induced cell-to-cell fusion (*A* and *C*) and cell numbers for each syncytium (*B* and *D*) (n > 15) were determined. (*E–J*) Vero-E6 cells transfected with OCLN or control siRNA were infected with SARS-CoV-2-mNG or rVSV-eGFP-S at an MOI of 0.1. After 1-h incubation, virus was removed, and the cell layer overlaid with 3% agarose with or without 5 μg/mL trypsin and Baf-A1 for 72 h. Images of cells (*E* and *H*) were captured (scale bars, 300 μm); cell numbers for each plaque (*F* and *I*) and virus titer (*G*, *J*, *K*, and *L*) were determined and analyzed. (*M* and *N*) 293FT-hACE2-TMPRSS2 cells transfected with OCLN or control siRNA were infected with SARS-CoV-2-mNG or rVSV-eGFP-S at an MOI of 0.01 for 1 h. After 1-h incubation, virus was removed and overlaid with 3% agarose with Baf-A1. Seventy-two h postinfection, images (*M*) were captured (scale bars, 300 μm), and cell numbers for each plaque (*N*) were determined.

SARS-CoV-2 uses two distinct pathways for cell entry: an endosome one facilitated by cathepsin L or cathepsin B (29) and a protease-mediated one located at the cell surface facilitated by the transmembrane serine protease 2 (TMPRSS2) and experimentally modulated by exogenous trypsin (30, 31). To determine whether the endosome pathway is involved in OCLN-mediated cell-to-cell transmission, Vero-E6 cells infected with rVSV-eGFP-S were treated with Catl inhibitor III (an inhibitor of cathepsin L) and Baf-A1 (a Na+/K+ ATPase pump inhibitor), which have been previously used to block SARS-CoV-2 entry from the endosome pathway (32–34). Both compounds dramatically inhibited syncytium formation and blocked virus-induced cell-to-cell fusion according to GFP signals and numbers of syncytia when compared to mock-treated control groups (Fig. 7 *A*–*D*).

To determine whether the cell surface entry pathway is also involved in OCLN-mediated SARS-CoV-2 cell-to-cell transmission, SARS-CoV-2 infected with Vero-E6 cells were treated with DMSO alone as a control or the mixture of trypsin and Baf-A1 and then overlaid with 3% agarose to block virus transmission via a cell-free way. As expected, trypsin treatment provided greater syncytial formation than in control cells after infection with either SARS-CoV-2-mNG or rVSV-eGFP-S virus (Fig. 7 *E* and *H*), consistent with a role for the cell surface pathway in virus entry. To measure the virus titer in the cells, the agarose–cell mixture was resuspended in PBS and subjected to three cycles of freeze–thaw to release the virus from cells. The released virus titer in the PBS was determined by the TCID_50_ assay. In contrast to cells that had not been treated with trypsin, there was no significant difference in the extent of cell fusion and virus yield between control siRNA–treated and specific OCLN siRNA–treated groups in the presence of trypsin (Fig. 7 *F*, *G, I*, and *J*), suggesting that trypsin exposure neutralized the effect of OCLN knockdown. Moreover, trypsin treatment significantly increased virus yield in the cells compared with the control group (Fig. 7 *K* and *L*).

To further confirm this result, we used SARS-CoV-2-mNG and rVSV-eGFP-S viruses to infect 293FT-hACE2-TMPRSS2 cells that stably express human ACE2 and TMPRSS2, which were reported to increase syncytium formation by accelerating cleavage of the S protein (30). As shown in Fig. 7 *M* and *N*, no differences in syncytium formation and infected cell numbers were observed between control siRNA and OCLN knockdown cells infected with either SARS-CoV-2-mNG or rVSV-eGFP-S virus. Taken together, these results demonstrate that the endosomal entry pathway is involved in OCLN-mediated cell-to-cell transmission rather than the cell surface entry pathway.

### OCLN Mediates SARS-CoV-2 Variants Cell-to-Cell Transmission.

**Fig. 8.**
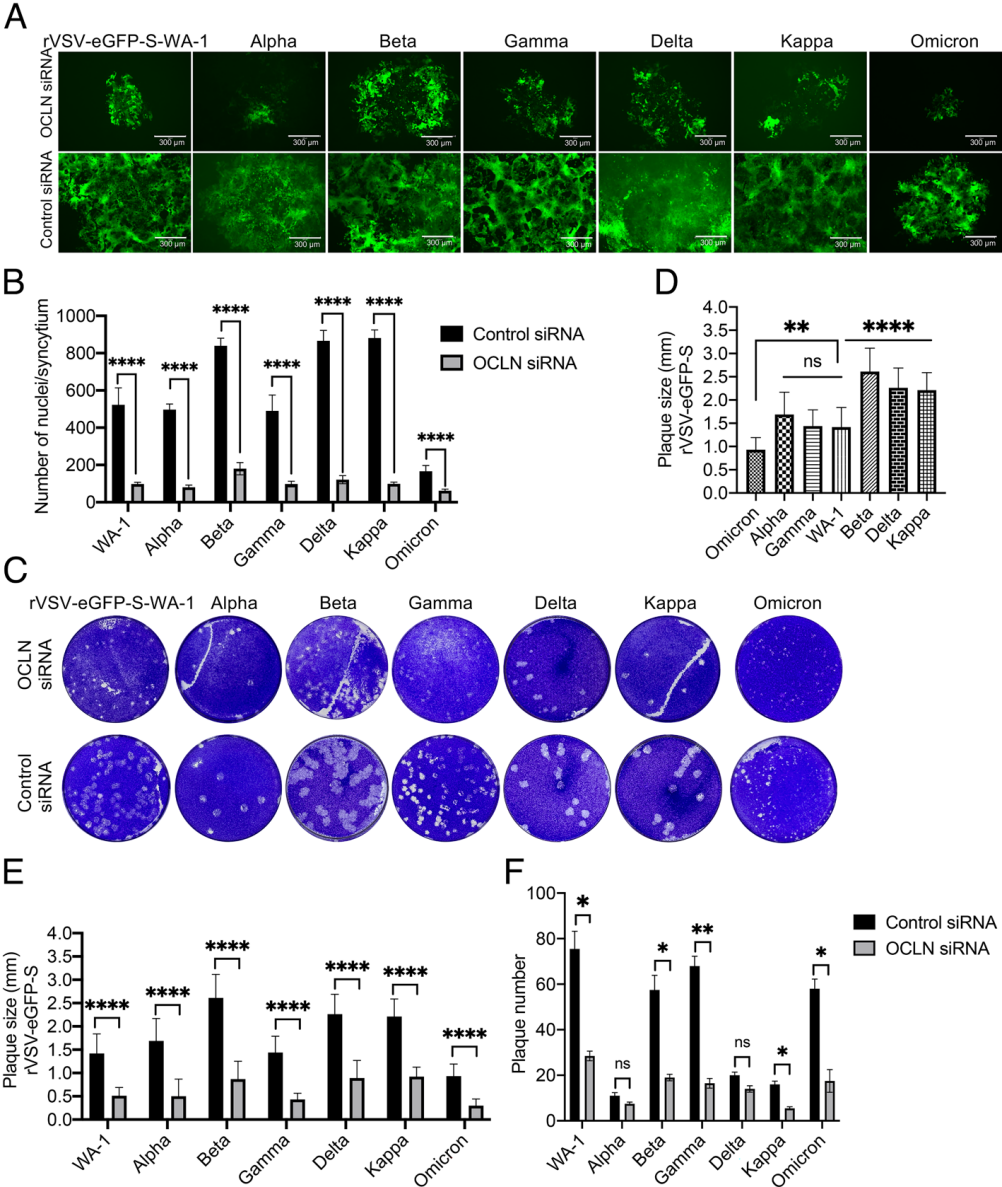
OCLN mediates SARS-CoV-2 variants cell-to-cell transmission. (*A* and *B*) A549-hACE2 cells were transfected with OCLN or control siRNA prior to infection with rVSV-eGFP-S variants at an MOI of 0.01 for 72 h. Virus infection was observed by GFP signals and images (*A*) were captured (scale bars, 300 μm). The numbers of nuclei for each syncytium (*B*) were determined. (*C–F*) A549-hACE2 cells were transfected with OCLN siRNA or control siRNA prior to infection with rVSV-eGFP-S variants at an MOI of 0.1 for 72 h. Plaque assays (*C*) were performed to determine virus transmission. Plaque sizes for nontreated groups (*D*) and for each virus after OCLN knockdown (*E*) were compared and analyzed, and plaque numbers for each virus after OCLN knockdown (*F*) were analyzed. Data analysis was performed with GraphPad Prism 9 software (ns, *P* > 0.05; ****P < 0.0001; **P < 0.01; and *P < 0.05).

To determine the role of OCLN on cell-to-cell transmission of SARS-CoV-2 variants, experiments were conducted to rescue replication-competent rVSVs expressing the spike of different SARS-CoV-2 variants including the alpha, beta, gamma, delta, kappa, and omicron BA2 strains, which were used to infect the A549-hACE2 cells. As shown in Fig. 8 *A* and *B*, OCLN knockdown significantly inhibited virus infection and S protein–mediated syncytium formation with each of the virus strains, which is consistent with our previous findings (Fig. 6 *A* and *B*). Interestingly, the alpha and gamma variants showed similar degrees of syncytium formation to the prototypic WA-1 spike, whereas beta, delta, and kappa variants displayed larger areas of syncytium according to plaque size (Fig. 8 *C* and *D*). In contrast, the omicron BA2 variant formed the smallest sized plaques relative to the prototypic WA-1 and other variants (Fig. 8 *C* and *D*). Again, OCLN knockdown led to significantly reduced plaque size in all tested viruses (Fig. 8*E*) and generally decreased plaque numbers in prototypic WA-1, beta, gamma, kappa, and omicron variants compared with the nontreated groups (Fig. 8*F*). Taken together, these findings indicate that OCLN is involved in cell-to-cell transmission of all SARS-CoV-2 forms and is mediated by the spike protein.

## Discussion

Viral spread via tight cell–cell contacts is considered to assist immune evasion from neutralizing antibodies (17, 35, 36) and also to diminish the effectiveness of antiviral drugs owing to the high local virus concentrations present (37, 38). Notably, many human viruses have been documented to use either cell-free particles or direct cell–cell spread or both mechanisms to contribute to effective virus survival and infection (39, 40). So far, a range of host factors have been reported to account for the cell-to-cell spread of influenza A virus, human metapneumovirus, and respiratory syncytial virus (41–43). SARS-CoV-2 also spreads efficiently via direct cell-to-cell transmission (15); however, the host factors involved and the underlying mechanisms remain unclear. In the present study, we identified OCLN as a host factor mediating SARS-CoV-2 internalization and cell-to-cell transmission. OCLN knockdown reduces SARS-CoV-2 infection and spread, while OCLN overexpression promotes both processes. We also observed similar outcomes when using replication-competent rVSVs expressing the spike proteins of prototypic and a range of SARS-CoV-2 variants of concern. Interestingly, viruses expressing the beta, delta, and kappa variants of the spike protein displayed enhanced cell-to-cell transmission compared with the WA-1 strain spike, which is consistent with previous reports (9, 44). However, alpha and gamma variants showed similar capacities to drive syncytium formation as the prototypic WA-1 strain. On the other hand, these variants have been reported to enhance syncytium formation when compared with the ancestral Wuhan-like and WA-1 strains where the spike protein alone was used to test capacity to drive cell fusion and syncytium formation (44, 45). These differences are probably due to our use of replication-competent rVSVs rather than the spike protein alone. Our finding that the omicron variant displayed diminished ability to generate syncytia and formed smaller plaques in A549-hACE2 cells than either prototypic or other SARS-CoV-2 variant strains strongly suggests that omicron has reduced ability to spread from cell-to-cell via the cathepsin-mediated endocytic pathway. The observation may explain why omicron BA.1 and BA.2 sublineages show reduced pathogenicity and transmissibility in hamsters (7, 46) and induce milder clinical symptoms in humans relative to the WA-1 and other SARS-CoV-2 variants of concern (47, 48).

Our experiments suggest that SARS-CoV-2 infection likely only results in degradation of OCLN without or little impacts on other TJ proteins, such as ZO-1 and Claudin-1 (Fig. 1*A*). The opposite occurs during infection of Vero-E6 cells by PEDV in which OCLN is the primary entry factor, but its expression becomes up-regulated rather than diminished (24). Normally, TJ proteins are in a highly dynamic state, undergoing continuous endocytosis and recycling back to the plasma membrane in a process that provides TJs’ plasticity and function (49, 50). Although it is not clear why SARS-CoV-2 infection down-regulates both the production and stability of the OCLN protein, both phenomena appear to be dependent on SARS-CoV-2 infection and replication since infection with UV-inactivated SARS-CoV-2 does not affect either OCLN distribution or expression (Fig. 1*G*). We are considering two possibilities. One is that the activation of signaling pathways observed in response to infection by SARS-CoV-2 and some other viruses, e.g., MERS-CoV and Ebola (51, 52), somehow influence OCLN. The second is that viral proteins hijack the protein recycle process associated with TJs. The underlying mechanisms of SARS-CoV-2 regulating OCLN in TJs need to be investigated in future studies.

Binding to only single cell surface protein is often insufficient for a virus to initiate efficient infection, and also depending on the target cell type or entry steps, viruses frequently rely on one or more coreceptors for entry. Several host factors have been demonstrated as potential coreceptors for SARS-CoV-2 infection. For example, tyrosine-protein kinase receptor UFO is critical for SARS-CoV-2 infection in H1299 pulmonary cells and human primary lung epithelial cells (53). Another study has identified the ACE2/ASGR1/KREMEN1 (ASK) receptor combination used by SARS-CoV-2 in different cell types through genomic receptor profiling (54). Additionally, metabotropic glutamate receptor subtype 2, a cellular receptor for rabies virus (55), has also been shown to be an internalization factor for SARS-CoV-2, SARS-CoV, and MERS-CoV (2). OCLN identified in this study is not only a TJ component but a target for proinflammatory cytokines (56) and a coreceptor for hepatitis C virus or coxsackievirus B3 (20, 22). Here, we reveal a role of OCLN as an internalization factor for SARS-CoV-2 entry and further demonstrate that its C-terminal is responsible for mediating this effect and for SARS-CoV-2 cell-to-cell spread (Fig. 4 *B* and *C*). SARS-CoV-2 S1 directly binds to hOCLN complexed with ACE2 (Fig. 3 *C* and *D*), suggesting that S1 binding to both ACE2 and OCLN is critical for initiating virus entry and subsequently replication and transmission. Interestingly, mOCLN shows similar functions as hOCLN to mediate SARS-CoV-2 internalization and cell-to-cell spread (*SI Appendix*, Fig. S5), suggesting SARS-CoV-2 infection in mice could induce severe pathology, especially in strains adapted to SARS-CoV-2 infection (57). Knockdown of OCLN in SARS-CoV-2permissive Vero-E6 and A549-hACE2 cells does not impact ACE2 expression (*SI Appendix*, Fig. S1 *B* and *C*) but significantly reduces virus infection, especially during the internalization stage and subsequent viral replication. In contrast, overexpression significantly enhances both virus internalization and replication but not virus binding. Hence, OCLN cannot strictly be considered as either a receptor or coreceptor for entry of SARS-CoV-2.

Our studies also reveal that the endosomal entry pathway rather than the cell surface membrane pathway is involved in OCLN-mediated cell-to-cell transmission of SARS-CoV-2. Treatment of infected cells with endosomal entry pathway inhibitors diminishes syncytium formation and cell-to-cell spread of SARS-CoV-2, leading to significantly reduced virus infection and replication (Fig. 7 *A*–*D*). However, trypsin treatment or presence of TMPRSS2 facilitated the cell surface pathway, which is not affected by OCLN knockdown, and no difference is observed on virus transmission between OCLN knockdown and mock groups. Therefore, the cell surface entry pathway is not involved in OCLN-mediated cell-to-cell transmission.

Finally, trypsin treatment significantly promotes SARS-CoV-2 cell-to-cell transmission (Fig. 7*E*), most likely because protease-mediated cell surface entry is more efficient than the endosomal entry as similar results to those observed here with SARS-CoV have been noted with porcine deltacoronavirus (58, 59). Interestingly, trypsin treatment has been reported to decrease virus titers in supernatants after SARS-CoV-2 infection (60). This is most likely because SARS-CoV-2 is more sensitive to trypsin treatment owing to having a unique furin cleavage site in its spike between S1 and S2 and that S1/S2 cleavage further promotes the exposure of the S2′ cleavage site to trypsin as reported for MERS-CoV (13). We speculate that cell-free SARS-CoV-2 may be subjected to overcleavage in the presence of trypsin in the medium and that will impair the stability of viral particles, thereby reducing virus infectivity and yield in cell culture supernatant. This phenomenon is also found in PEDV where, again, there is reduced infectivity after pretreatment with trypsin (61). In the present study, trypsin treatment facilitated cell-to-cell transmission and increased virus yield in cells (Fig. 7 *K* and *L*), suggesting that cell-to-cell transmission helps retain infectivity of viral particles compared with cell-free transmission. Therefore, to avoid protease-mediated overcleavage, cell-to-cell transmission is the best way to keep the virus high infectivity and transmissibility.

This study demonstrates OCLN as a crucial host factor participating in SARS-CoV-2 cell-to-cell transmission. Our results not only reveal important mechanistic details of the role of OCLN in the internalization step of the SARS-CoV-2 entry and also provide solid evidence of OCLN-mediated cell-to-cell transmission of authentic SARS-CoV-2 and other variants of concern so far identified.

## Materials and Methods

Cell lines, viruses, RNAi, hamster study, plasmids, OCLN KO cell line, confocal assay, immunofluorescence assay, western blot assay, RT-qPCR assay, co-IP assay, GST pull-down assay, plaque assay, pharmacological inhibitor assay, and statistical analysis are described in *SI Appendix*, *Materials and Methods*.

### Virus Binding Assay.

Vero-E6, A549-hACE2, and Caco-2 cells in a 24-well plate were transfected with the OCLN siRNA for 72 h, and the plate was transferred onto the ice for 20 min. Then, the cells were infected with SARS-CoV-2 at an MOI of 1 at 4 °C for 1 h. Unattached viruses were removed through washing three times with cold PBS, and cells were treated with 1 ml TRIzol to prepare RNA samples. Viral RNA was detected by RT-qPCR targeting the N gene.

### Virus Internalization Assay.

After 72-h OCLN siRNA transfection, the plate was transferred onto the ice for 20 min. Cells were infected with SARS-CoV-2 at an MOI of 1 at 4 °C for 1 h. Then, the cells were washed three times with cold PBS to remove unbound viruses. The plate was transferred to 37 °C for 1 h to allow virus internalization. Then, the bound viruses were removed by washing three times with acidic buffer (50 mM glycine and 100 mM NaCl, pH 3.0) and then trypsinized to remove SARS-CoV-2. This treatment has been approved to efficiently remove the bound SARS-CoV-2 virus on Vero-E6 cells (2). The cells were lysed, and RNA prepared with 1 mL TRIzol. Viral RNA was detected by RT-qPCR targeting the N gene.

### Plaque Assay.

A549-hACE2 or Vero-E6 cells were transfected with the OCLN siRNA for 72 h and then infected with SARS-CoV-2 or rVSV-eGFP-S at an MOI of 0.01 at 37 °C for 1 h. The cells were then washed with PBS to remove the unbound viruses and covered with 3% low-melt agarose gel. After the top agar is solidified, the plate is inverted and incubated at 37 °C. After 72 h of incubation, the cells were stained with crystal violet. Plaque numbers were counted, and relative plaque size was determined with ImageJ software.

### Ethics Statement.

All research related to SARS-CoV-2 viruses was approved by the Institutional Biosafety Committee at the University of Missouri and performed in the BSL-3 laboratory at the University of Missouri Laboratory for Infectious Disease Research (LIDR). The hamsters infected with the SARS-CoV-2 virus were approved by the Institutional Animal Care and Use Committee at the University of Missouri and was performed in Biosafety Level 3 animal facilities at the LIDR.

## Supplementary Material

Appendix 01 (PDF)

## Data Availability

All study data are included in the article and/or *SI Appendix*.
