# Supplementary material for: Tight junction protein occludin is an internalization factor for SARS-CoV-2 infection and mediates virus cell-to-cell transmission
Source: Proc Natl Acad Sci U S A. 2023 Apr 17;120(17):e2218623120. doi: 10.1073/pnas.2218623120 (PMC10151465; doi:10.1073/pnas.2218623120)
Supplement: Supplementary file 1 — Appendix 01 (PDF) [file pnas.2218623120.sapp.pdf]

PNAS

Supporting Information for

Tight junction protein occludin is an internalization factor for SARS-CoV-2 infection and mediates virus cell-to-cell transmission

Jialin Zhang[a,b], Wenyu Yang[a,b], Sawrab Roy[a,b], Heidi Liu[a,b], R. Michael Roberts[c,d], Liping Wang[a,b], Lei Shi[a,b], Wenjun Ma[a,b,*]

*To whom correspondence should be addressed: Wenjun Ma

Email: wma@missouri.edu

This PDF file includes:

Supporting text
Figures S1 to S5
SI References

Supplementary Materials and Methods

Cell lines

Vero-E6 cells (ATCC, CRL-1586), Caco-2 cells (kindly provided by Dr. Feng Li at University of Kentucky), and BSR-T7/5 cells (kindly provided by Dr. Karl-Klaus Conzelmann) were cultured in Dulbecco's modified Eagle's medium (DMEM) supplemented with 10% fetal bovine serum (FBS) and 1% penicillin/streptomycin at 37°C in a 5% CO_2 incubator. 293FT cells expressing human ACE2 and Transmembrane Serine Protease 2 (TMPRSS2) (293FT-hACE2-TMRPSS2) (a kind gift from Dr. Marc Johnson at University of Missouri-Columbia) were maintained in DMEM supplemented with 10% FBS, MEM vitamin, L-glutamine, sodium pyruvate, non-essential amino acids and selected with puromycin (1 μg/mL) and hygromycin (200 μg/mL) (1). A549-hACE2 cell line expressing human ACE2 was maintained in DMEM supplemented with 10% FBS and selected with 0.5 μg/mL of puromycin (InvivoGen, a549-hace2)

Viruses

SARS-CoV-2 reverse genetic system was kindly provided by Dr. Pei-Yong Shi at University of Texas Medical Branch. SARS-CoV-2-mNG virus expressing the mNeonGreen gene was rescued on Vero-E6 cells in a BSL-3 facility according to an appropriate protocol (2). The SARS-CoV-2 USA-WA1/2020 strain was acquired from BEI Resources (Manassas, VA, USA) and kindly provided by Drs. Jeff Adamovicz and Jeff Whyte at University of Missouri. Plasmids encoding codon-optimized full-length Spike of the Wuhan-1 strain (VRC7480), Alpha (B.1.1.7), Beta (B.1.351), Gamma (P.1), Delta (B.1.617.2), Kappa (B.1.617.1) and Omicron (BA.2) were kindly provided by Dr. Tongqing Zhou at the Vaccine Research Center, NIAID/NIH. Vesicular stomatitis virus (VSV) reverse genetic system was kindly provided by Dr. Sean Whelan at Washington University in St. Louis. rVSV-eGFP-SARS-CoV-2-S virus (rVSV-eGFP-S) was rescued on BSR-T7/5 cells and passaged on Vero-E6 cells. Recovery of the rVSV-eGFP-S virus was performed as described previously (3). Briefly, BSR-T7/5 cells in a 6-well plate were infected with the vaccinia virus MVA-T7 (kindly provided by Dr. Bernard Moss at NIH) at 37°C for 1 h and subsequently transfected with 7 μg of mixed plasmids of VSV-eGFP-SARS-CoV-2, N, P, and L with a 5:3:5:1 ratio. Forty-eight hours posttransfection, the supernatants were filtered to remove the vaccinia virus and passaged on Vero-E6 cells. SARS-CoV-2-mNG and rVSV-eGFP-S virus stocks were prepared and titrated on Vero-E6 cells and stored at -80°C. The PEDV strain was isolated from pigs by Dr. Ma's laboratory in 2013.

Hamster study

Nine 6 to 8-weeks-old male and female Syrian hamsters were divided into 2 groups and held in the BSL3 animal facility at the University of Missouri Laboratory for Infectious Disease Research. Six hamsters were intranasally infected with 10^6 $TCID_{50}$ of the SARS-CoV-2/USA-WA1/2020 virus per animal and 3 hamsters were mock-infected with PBS as controls. Clinical signs were monitored daily. Three infected and control hamsters were euthanized and necropsied on day 3 postinfection and the remaining 3 infected hamsters were necropsied on day 5 postinfection. During necropsy, the lung was collected from each animal for western blot assay to determine levels of OCLN.

Plasmids

SARS-CoV-2 S1 subdomain (aa 14-685) was amplified and cloned into pCAGGS vector with a HA tag or a His tag in the C-terminal to generate pCAGGS-S1-HA or pCAGGS-S1-His plasmid. The human OCLN (hOCLN) gene was amplified from RNA of 293T cells and cloned into pCAGGS vector with a Flag tag in the C-terminal or pCold-GST vector (Takara, 3372), resulting in pCAGGS-hOCLN-flag and pCold-GST-hOCLN plasmids. The hOCLN gene was cloned into pLenti-CMV/TO-eGFP-Puro plasmid by using BamHI/XbaI to generate the lentiviral plasmid pLenti-CMV/TO-hOCLN-Puro to overexpress OCLN on Vero-E6 cells or A549-hACE2 cells. Mouse occludin (mOCLN) was amplified from RNA extracted from mouse lung tissue and cloned into pCAGGS vector with a Flag tag in the C-terminal to generate a plasmid, named the pCAGGS-mOCLN-Flag. hOCLN/ΔN (deletion of aa 1-63), hOCLN/ΔC (deletion of aa 267-522), hOCLN/ΔEL1 (deletion of aa 94-128), and hOCLN/ΔEL2 (deletion of aa 200-238) as described previously (4) were amplified by using overlap-PCR and cloned into pCAGGS vector with a Flag tag in the C-terminal to generate

pCAGGS-hOCLN/ΔN-Flag, pCAGGS-hOCLN/ΔC-Flag, pCAGGS-hOCLN/ΔEL1-Flag, and pCAGGS-hOCLN/ΔEL2-Flag plasmids, respectively.

OCLN knockout cell line

The guide sequence oligonucleotides including the BsmBI restriction site overhangs (gRNA1: 5'-CACCGAGTGTAGGCTACCCTTATGG-3', 5'-AAACCCATAAGGGTAGCCTACACTC-3'; gRNA2: 5'-CACCGGATAATAGTGAGCGCTATCC-3', 5'-AAACGGATAGCGCTCACTATTATCC-3') were designed and cloned into the lentiCRISPRv2-blast vector (Addgene, 98293) to generate lentiCRISPRv2-blast-gRNA. 293T cells in 100-mm dishes were transfected with plasmids using Lipofectamine 3000 according to the manufacturer's instructions to prepare lentivirus. Briefly, 10 µg of lentiCRISPRv2-blast-gRNA, 8 µg of psPAX2 (Addgene, 12260), and 5 µg of pCMV-VSV-G (Addgene, 8454) were transfected into 293T cells. The medium was changed at 6 h posttransfection and at 48 h the supernatant was collected, filtered through a 0.45 µm PES filter, and concentrated with Amicon Ultra-4 centrifugal filters. Vero-E6 cells in 6-well plates were infected with the concentrated lentivirus at 37°C for 6 h. Then, the virus was removed, and 2 ml of fresh medium was added. After 48 h of incubation, the medium was replaced with the selective medium containing 6 µg/ml of Blasticidin S HCl (ThermoFisher, A1113903). After 7 days of selection, cells were subjected to single-clone selection using serial dilutions in a 96-well plate. After 3 days of incubation, wells containing single cells were marked and knockout cells were identified by the Guide-it Genotype Confirmation Kit (Takara, 632611), and further confirmed by IFA and western blot. Positive colonies were transferred into 6-well plates for amplification and further experiments.

Confocal assay

Vero-E6 cells in a 35-mm glass dish were infected with SARS-CoV-2-mNG or rVSV-eGFP-S virus at an MOI of 0.1. The cells were then fixed with 4% paraformaldehyde for 20 mins and permeabilized with 0.25% Triton X-100 for 15 min followed by blocking with 5% non-fat milk for 1 h at room temperature. Rabbit anti-OCLN antibody (1:200, Invitrogen, 71-1500), mouse anti-ZO-1 antibody (1:250, Invitrogen, 339100), rabbit anti-claudin antibody (1:1000, Abcam, ab211737) were added and incubated at 4°C overnight, followed by incubating with an Alexa Fluor™ 647-conjugated goat anti-rabbit IgG (H+L) cross-adsorbed secondary antibody (A-21244, Invitrogen) or an Alexa Fluor™ 647-conjugated goat anti-mouse IgG (H+L) cross-adsorbed secondary antibody (A-21235, Invitrogen) at 37°C for 1 h, respectively. Nuclei were stained with 4',6-diamidino-2-phenylindole (DAPI).

For the ACE2 and OCLN colocalization assay, Vero-E6 cells were infected with SARS-CoV-2/WA1 strain at an MOI of 0.1, and then fixed with methanol/acetone (3:2) at -20°C for 30 min. After blocking with 5% non-fat milk, the cells were incubated with a mouse anti-ACE2 antibody (1:200, Invitrogen, MA5-31395) or a rabbit anti-occludin antibody (1:200, Invitrogen, 71-1500) at 4°C overnight, followed by exposure to secondary antibodies as described above. Images were captured with a Leica SP8 spectral confocal microscope.

Immunofluorescence assay

Vero-E6 cells in 24-well plates were infected with the SARS-CoV-2-mNG virus at an MOI of 0.01 at 37°C for 1 h. Then, the cells were washed three times with PBS and fresh DMEM with 2% FBS was added. After 48 h, the cells were fixed with methanol/acetone (3:2) at -20°C for 30 min and non-specific antibody binding blocked with 2% BSA for 2 h at room temperature. The cells were stained with an OCLN monoclonal antibody diluted 1:250 in 0.1% BSA (Invitrogen, 33-1500) at 4°C overnight, and then stained with the Alexa Fluor 594-conjugated goat anti-mouse IgG (H+L) at a dilution of 1:500 (Jackson ImmunoResearch Laboratories, 115-585-003) at 37°C for 2 h.

For the siRNA assays, the cells were stained with ZO-1 (1:250, Invitrogen, 339100) or Claudin-1 (1:1000, Abcam, ab211737) antibody after fixation and blocking at 4°C overnight, followed by incubating with an Alexa Fluor 594-conjugated goat anti-mouse IgG (H+L) (Jackson ImmunoResearch Laboratories, 115-585-003) and Alexa Fluor Plus 594 labeled goat anti-rabbit IgG (H+L) antibody at 37°C for 2 h, respectively. Nuclei were stained with DAPI. Images were acquired with the EVOS™ M5000 imaging system.

Western blot assay

Vero-E6 cells were infected with SARS-CoV-2 at an MOI of 0.01. Cells were harvested at indicated time points (24 h and 48 h) and lysed with RIPA on ice for 30 min. Lungs were collected from three mock-infected hamsters at day 3 postinfection and three hamsters infected with the SARS-CoV-2/WA1 virus at day 3 and 5 postinfection, respectively, for the western blot assay. Lung tissues (100 mg) from each animal were mixed with 1 ml CelLytic™ M buffer (C2978, Sigma-Aldrich) containing protease inhibitors and homogenized. The supernatant was collected after centrifugation at 12,000 × *g* for 30 min. The supernatants were mixed with 4 X loading buffer and boiled for 20 mins to prepare samples for electrophoresis on a 10% SDS-PAGE gel. After transferring protein to the PVDF membrane, the membrane was blocked with 5% non-fat milk in PBS for 1 h at room temperature. Then, the membrane was incubated with an OCLN monoclonal antibody (1:1,000, Invitrogen, 33-1500), an anti-SARS-CoV-2-nucleocapsid (N) rabbit monoclonal antibody (1:2,000, Genuin Biotech LLC, VYN7), and an anti-GAPDH mouse monoclonal antibody (1:1,000, Santa Cruz, sc-166545) at 4°C overnight, followed by incubation of the HRP-conjugated goat anti-rabbit IgG (H+L) (1:10,000, Invitrogen, A16096) or HRP-conjugated goat anti-mouse IgG (H+L) (1:10,000, Invitrogen, A16066) for 1 h at room temperature. Chemiluminescent detection was performed by using Pierce™ ECL Western blot Substrate (Thermo Scientific, 32106).

RT-qPCR

The OCLN mRNA level was determined after SARS-CoV-2 infection. The cellular RNA was prepared and extracted with 1 mL TRIzol (Invitrogen, 15596026) according to the manufacturer's instructions. RT-qPCR was performed by using Invitrogen™ SuperScript™ III Platinum™ SYBR™ Green One-Step RT-qPCR Kit (Invitrogen, 11-736-051) according to the manufacturer's instructions under the following conditions: reverse transcription (50°C for 5 min; 95°C for 2 min) for one cycle; PCR (95°C for 3 s; 60°C for 30 s) for 40 cycles with a CFX96 Touch Real-Time PCR System (Bio-Rad). Primers targeting green monkey OCLN gene and human OCLN gene were monkey OCLN-F: GACTTCAGGCAGCCTCGTTAC; monkey OCLN-R: GCCAGTTGTGTAGTCTGTTTCA; and human OCLN-F: ACTTCAGGCAGCCTCGTTAC; human OCLN-R: GCCAGTTGTGTAGTCTGTCTCA. Primers for monkey β-actin, human ACE2 and human 28S rRNA genes were used as described previously (5). Primers targeting green monkey ACE2 gene were monkey ACE2-F: TGGGACTCTGCCATTTACTTAC; and monkey ACE2-R: CCCAACTATCTCTCGCTTCATC. Primers (2019-nCoV_N2-F/2019-nCoV_N2-R) and probe (2019-nCoV_N2-P) targeting SARS-CoV-2 N were used as CDC recommended to detect viral RNA (https://www.cdc.gov/coronavirus/2019-ncov/lab/rt-pcr-panel-primer-probes.html). RT-qPCR was performed by using qScript® XLT One-Step RT-qPCR ToughMix® (Quantabio, 95132-02K) according to the manufacturer's instructions under the following conditions with some modifications: reverse transcription (50°C for 10 min; 95°C for 3 min) for one cycle; PCR (95°C for 3 s; 55°C for 30 s) for 45 cycles with a CFX96 Touch Real-Time PCR System (Bio-Rad). Relative quantification was performed by the cycle threshold (ΔΔCt) method.

Co-immunoprecipitation

293T cells were co-transfected with pCAGGS-SARS-CoV-2-S1-HA, pCAGGS-hACE2-HA, and pCAGGS-OCLN-flag, or pCAGGS empty vector using lipofectamine 3000 (Invitrogen, L3000075) according to the manufacturer's instructions. Seventy-two hours posttransfection, the cells were washed three times with cold PBS and lysed with Pierce™ IP Lysis Buffer (Thermo Scientific, 87787) on ice for 30 min. The supernatant was collected and mixed with 25 μl of pre-washed Pierce™ Protein G Magnetic Beads (Thermo Scientific, 88848) for 4 h at 4°C on a tube revolver rotator (Thermo Scientific, 11676341). The beads were removed with a magnetic stand and an anti-HA antibody (Abcam, ab9110) was added to the supernatant and incubated overnight at 4°C. Pre-washed Protein G Magnetic Beads (25 μl) were added to the sample and incubated at 4°C for 4-6 h. After five times washing with cold TBST, the magnetic beads were mixed with the loading buffer and boiled for 15 min to prepare the sample for SDS-PAGE.

GST pull-down assay

293T cells were transfected with the pCAGGS-S1-His plasmid. At 60 h post-transfection, cells and supernatant were collected, and S1 protein was purified with a His tag by using ProBond Purification System (ThermoFisher, K850-01). pCold-GST-hOCLN and pCold-GST empty plasmids

were transformed into *E. coli* BL21 (DE3) competent cells and induced with IPTG, respectively. GST and GST-tagged hOCLN were purified by glutathione agarose according to the manufacturer's instructions. In brief, IPTG-induced bacteria cultures were harvested and resuspended with cold PBS, followed by mild sonication on ice. The supernatant containing soluble proteins were collected after centrifugation at 12,000 × *g* for 30 min, then mixed with the resin and incubated for 4 h at 4°C with purified SARS-CoV-2 S1 protein. The resin was washed five times with cold PBS and incubated at 4°C for 4 h with the lysates of the HEK293T cells transfected with pCAGGS-S1-His. After five times washing with cold PBS, the bound proteins were detected by western blot by using a mouse anti-His tag monoclonal antibody (1:1,000) and a GST Tag monoclonal antibody (1:1,000).

Pharmacological inhibitors

Vero-E6 cells were infected with rVSV-eGFP-S virus at an MOI of 0.01 for 1 h at 37°C. Then, the cells were washed three times with PBS, and the medium for the plaque assay containing 50 nM Bafilomycin (Baf-A1, Abcam, ab120497) or 5 μM Cathepsin L inhibitor III (Sigma, 219427-5mg) was overlaid onto the cells, respectively. After 48 h of incubation, the GFP signal was observed, and images were acquired with the EVOS™ M5000 imaging system. For virus entry assay, Vero-E6 cells were pre-treated with EIPA or rottlerin at 50 μM and 10 μM, respectively at 37°C for 1 h, then infected with SARS-CoV-2 at an MOI of 1 at 4°C for 1 h. The infected cells were washed three times with cold PBS to remove unbound viruses. The plate was transferred to 37°C for 1 h to allow virus entry. The bound viruses were then removed by washing three times with acidic buffer (50 mM glycine, 100 mM NaCl, pH 3.0), and trypsinized to remove SARS-CoV-2. Viral RNA was detected by RT-qPCR targeting the N gene after RNA was extracted with 1 ml TRIzol.

Statistical analysis

Data were analyzed by using a Student's t test, and values are indicated with mean ± SD. All data are representative of at least three independent experiments. Data analysis was performed with the GraphPad PRISM 9 software (ns, $p > 0.05$; ****$p < 0.0001$; ***$p < 0.001$; **$p < 0.01$; *$p < 0.05$).

Supplemental Figures

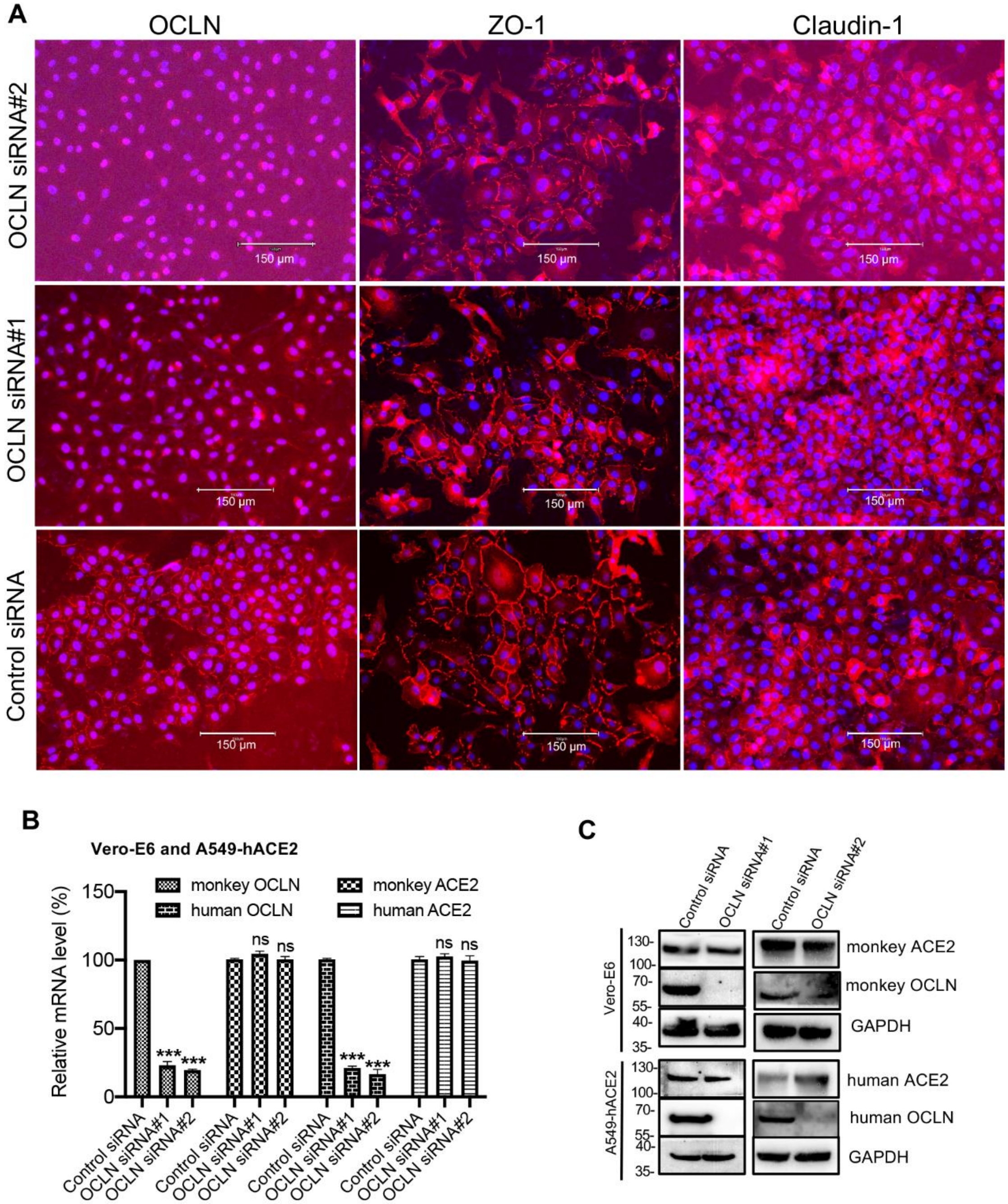

Fig. S1. OCLN knockdown has no effect on ACE2 expression. (*A*) Vero-E6 cells were transfected with two OCLN-specific siRNAs for 72 h, and IFA was performed to detect OCLN, ZO-1 and Claudin-1 expression (scale bars, 150 µm). (*B*) RT-qPCR was used to determine OCLN and ACE2 mRNA level after siRNAs treatment for 72 h. The results were representative of three independent experiments. (*C*) Western blot was used to determine OCLN and ACE2 expression after siRNAs treatment for 72 h.

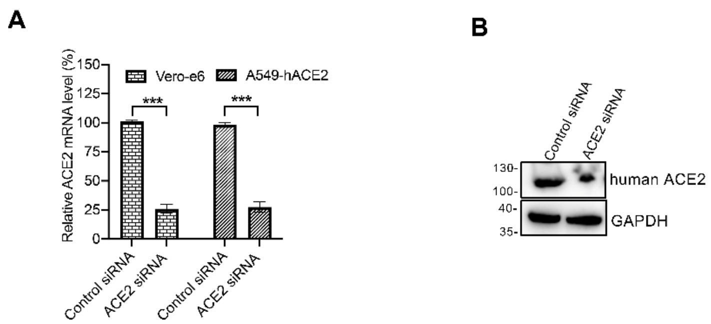

Fig. S2. ACE2 knockdown by ACE2-specific siRNA. (*A*) RT-qPCR was used to determine ACE2 knockdown after siRNA transfection in Vero-E6 cells and A549-hACE2 cells for 72 h. The results were representative of three independent experiments. (*B*) ACE2 knockdown in A549-hACE2 cells was determined by western blot.

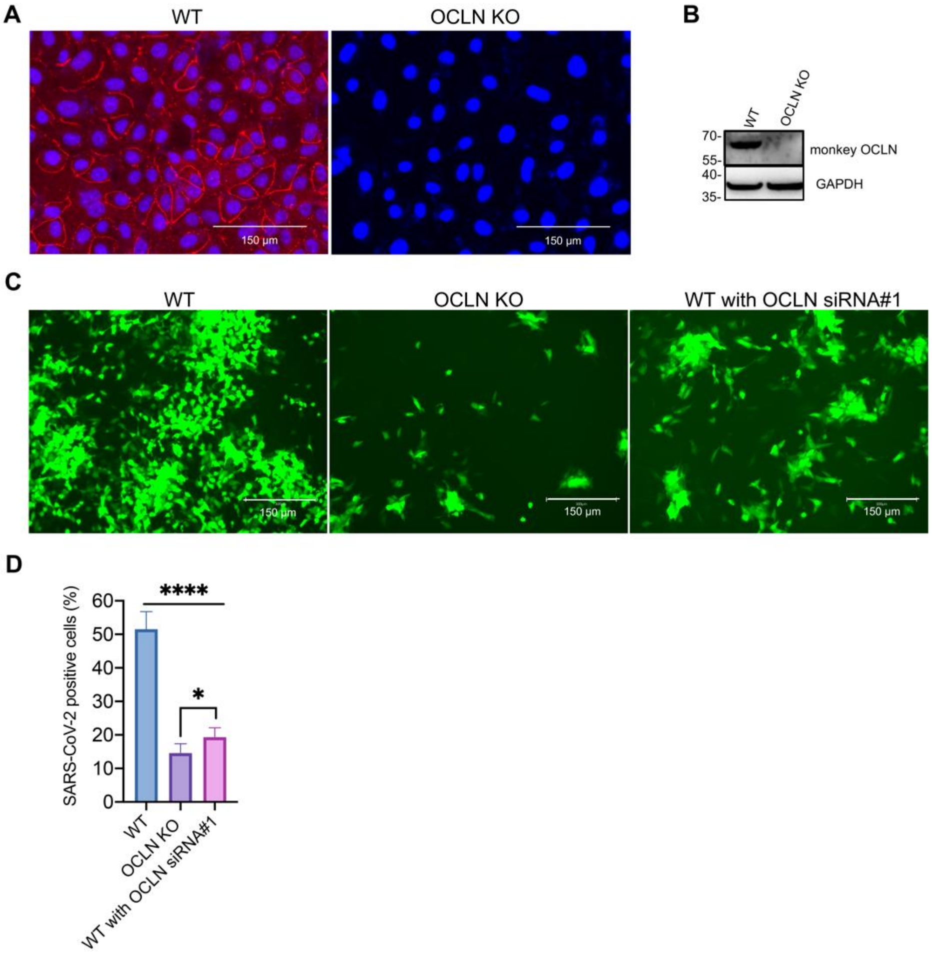

Fig. S3. Generation of Vero-E6 OCLN KO cell line. (*A*) OCLN expression was detected by IFA and (*B*) OCLN KO cells were confirmed by western blot by using an OCLN antibody. Wild type Vero-E6 was used as a positive control (scale bars, 150 µm). (*C*) Wild-type Vero-E6, wild-type Vero-E6 cells transfected with OCLN siRNA#1, and OCLN KO Vero-E6 cells were infected with SARS-CoV-2-mNG virus at an MOI of 0.01. At 24 h postinfection, imagines were captured (scale bars, 150 µm) and (*D*) Ten different fields for each group were captured and GFP-positive cells were analyzed with ImageJ software.

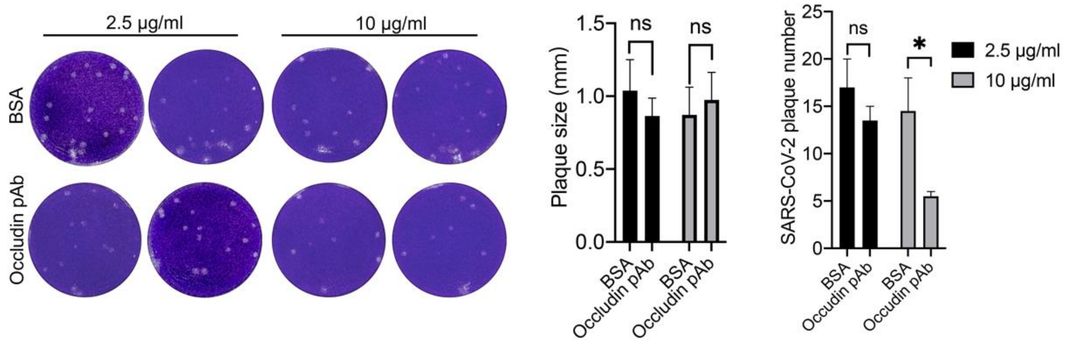

Fig. S4. OCLN polyclonal antibody blocking assay. Vero-E6 cells were incubated with BSA or an OCLN polyclonal antibody at indicated concentrations at 37°C for 1 h. Then, cells were infected with SARS-CoV-2 at an MOI of 0.001, and plaque assays were performed. Plaque number and plaque size were determined 72 h postinfection. The results were representative of three independent experiments.

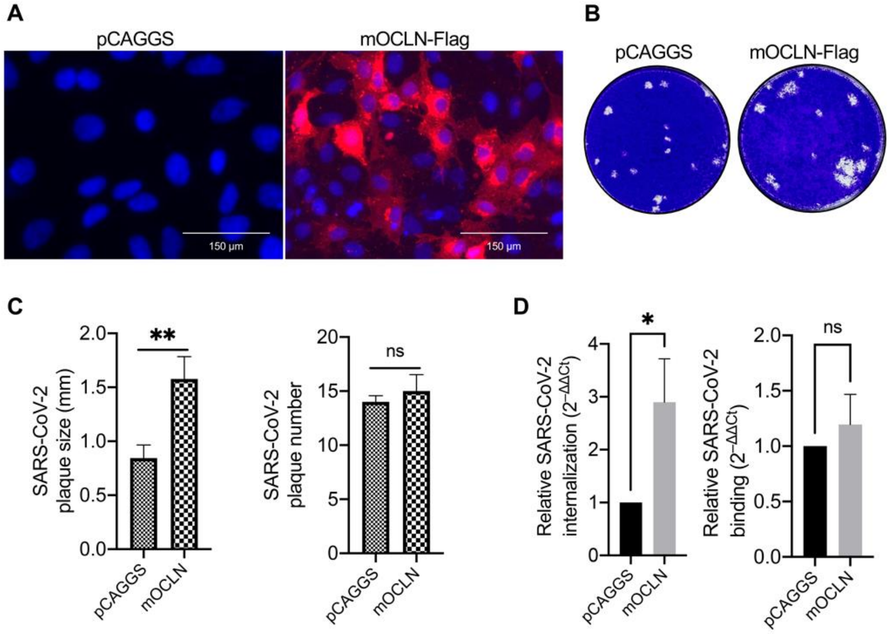

Fig. S5. Expression of mOCLN in Vero-E6 OCLN KO cells increases SARS-CoV-2 cell-to-cell transmission and internalization. (*A*) mOCLN was transfected to Vero-E6 OCLN KO cells and its expression production was detected by IFA with an anti-Flag antibody (scale bars, 150 μm). (*B*) Plaque assays were used to determine the effect of mOLCN expression on virus spread by measuring (*C*) plaque size and plaque number. (*D*) Virus internalization and virus binding were determined by RT-qPCR after mOCLN expression. The results were representative of three independent experiments.

Supplementary References

1. M. C. Johnson *et al.*, Optimized Pseudotyping Conditions for the SARS-COV-2 Spike Glycoprotein. *J Virol* **94** (2020).
2. X. Xie *et al.*, An infectious cDNA clone of SARS-CoV-2. *Cell host & microbe* **27**, 841-848. e843 (2020).
3. J. B. Case *et al.*, Neutralizing antibody and soluble ACE2 inhibition of a replication-competent VSV-SARS-CoV-2 and a clinical isolate of SARS-CoV-2. *Cell host & microbe* **28**, 475-485. e475 (2020).
4. S. Liu *et al.*, The second extracellular loop dictates Occludin-mediated HCV entry. *Virology* **407**, 160-170 (2010).
5. J. Wang *et al.*, SARS-CoV-2 uses metabotropic glutamate receptor subtype 2 as an internalization factor to infect cells. *Cell discovery* **7**, 1-16 (2021).
